# A brain-accessible peptide modulates stroke inflammatory response and neurotoxicity by targeting BDNF-receptor TrkB-T1 specific interactome

**DOI:** 10.7150/thno.111272

**Published:** 2025-03-21

**Authors:** Lola Ugalde-Triviño, Gonzalo S. Tejeda, Gema M. Esteban-Ortega, Margarita Díaz-Guerra

**Affiliations:** 1 Instituto de Investigaciones Biomédicas Sols-Morreale (IIBM), Consejo Superior de Investigaciones Científicas-Universidad Autónoma de Madrid, Madrid 28029, Spain.; 2 Institute of Molecular, Cell and Systems Biology, College of Veterinary Medical and Life Science, University of Glasgow, Glasgow, UK.

**Keywords:** cell-penetrating peptides, excitotoxicity, inflammatory response, interactome, neurodegeneration, neuroprotection

## Abstract

Glia reactivity, neuroinflammation and excitotoxic neuronal death are central processes to ischemic stroke and neurodegenerative diseases, altogether a leading cause of death, disability, and dementia. Given the high incidence of these pathologies and the limited efficacy of current treatments, developing brain-protective therapies that target both neurons and glial cells is a priority. Truncated neurotrophin receptor TrkB-T1, a protein produced by these cell types, plays relevant roles in excitotoxicity and ischemia. We hypothesized that interactions mediated by isoform-specific TrkB-T1 sequences might contribute to neurotoxicity and/or reactive gliosis, thus representing potential therapeutic targets.

**Methods:** We designed cell-penetrating peptides containing TrkB-T1 isoform-specific sequences to: 1) characterize peptide delivery into rat primary cortical cultures and mice brain cortex; 2) isolate and identify the isoform interactome in basal and *in vitro* excitotoxic conditions; 3) analyze peptide effects on neuroinflammation and neurotoxicity using primary cultures subjected to excitotoxicity or *in vivo* in a mouse model of ischemia*.*

**Results:** We identify here the TrkB-T1-specific interactome, poorly described to date, and demonstrate that interference of these protein-protein interactions using brain-accessible TrkB-T1-derived peptides can reduce reactive gliosis and decrease excitotoxicity-induced damage in cellular and animal models of stroke, where treatment reduces the infarct volume in male and female mice.

**Conclusions:** The crucial role of TrkB-T1 in modulating microglia and astrocyte reactivity indicates that isoform-derived peptides hold promise for the development of therapies for human stroke and other excitotoxicity-associated pathologies.

## Introduction

Stroke is a leading cause of death, disability, and dementia. Ischemic stroke, which accounts for approximately 85% of total cases, results from the blockage of a cerebral blood vessel, leading to reduced nutrient and oxygen levels in the affected area, culminating in neuronal death and irreversible brain damage. After an insult, two damaged areas can be distinguished, the infarct core and the penumbra. The infarct contains irreversible damaged tissue while the penumbra is functionally impaired but metabolically active. Consequently, the penumbra has become a neuroprotection target, although, if no therapy is applied, neurons can suffer secondary death mainly due to excitotoxicity. This process results from overactivation of glutamate receptors, particularly N-methyl-D-aspartate receptors (NMDARs), followed by massive influx of Ca^2+^ ions and subsequent disruption of ion homeostasis. Another contribution to ischemic stroke pathophysiology is the inflammatory response 1. Resident immune cells, including astrocytes and microglia, result activated in the acute phase, preceding passage of circulating immune cells to injured tissue across the damaged blood-brain barrier (BBB). Reactive astrogliosis, a process occurring in the peri-infarct environment, implies a dramatic cellular transformation that increases proliferation/migration, modifies morphology and leads to upregulation of genes such as *Gfap,* encoding glial fibrillary acidic protein 2. A subtype of reactive astrocytes loses homeostatic properties and experiences a pro-inflammatory gain of function with detrimental effects on neuronal and oligodendrocyte survival 3-5. Neuronal function is also regulated by microglia through neurotrophic factors release or specialized junctions formed by microglia processes with synaptic elements or neuronal bodies 6. Interestingly, a cross-talk between astrocyte and microglia activation is established in the CNS and, for example, neurotoxic reactive astrocytes are induced by microglia secretion of cytokines such as complement component 1q (C1q) 7.

Mechanisms underlying excitotoxicity and reactive gliosis are still largely undefined although they might unveil novel targets for stroke therapy. Conventional pharmacological thrombolysis and mechanical thrombectomy have significant limitations and benefit only a small proportion of patients 8. Therefore, we need to identify new candidates and develop strategies ideally targeting the different pathological processes and cell types involved in stroke. Signaling by brain-derived neurotrophic factor (BDNF)-binding to its high-affinity transmembrane receptor, tropomyosin-related kinase B (TrkB), becomes profoundly aberrant in stroke 9,10. In physiological conditions, BDNF is produced by multiple cells, including neurons and glia 11, and promotes neuronal maturation, differentiation, survival and synaptic function, among other processes. Different TrkB isoforms are produced by gene *Ntrk2* alternative splicing 12, full-length TrkB (TrkB-FL) and truncated TrkB-T1 being the major murine cortex isoforms 13. TrkB-T1 is the predominant isoform in the adult nervous system where it is mostly expressed by astrocytes and, secondarily, neurons. In contrast, TrkB-FL is almost exclusively expressed in neurons 14. BDNF-binding to TrkB-FL induces receptor dimerization, increased tyrosine kinase (TK) activity and transphosphorylation, leading to activation of interconnected signaling pathways that, altogether, stimulate processes central to CNS functioning. These pathways include the activation of prosurvival transcription factors (TFs) such as cAMP response element-binding protein (CREB) 15 and myocyte enhancer factor 2 (MEF2) 16,17, which regulate a wide array of genes, including those encoding BDNF 18-20, TrkB 21 and certain NMDAR subunits 22. In neurons, BDNF can also bind to TrkB-T1, truncated isoform lacking the TK region and traditionally considered as a dominant-negative receptor inhibiting BDNF-signaling 23. TrkB-T1 expressed in hippocampal astrocytes also participates in fine-tuning of neurotrophin-signaling by BDNF storage and translocation after BDNF/TrkB-T1 internalization 24.

In response to BDNF, TrkB-T1 also regulates important astrocyte functions including calcium release from intracellular stores 25, glycine 26 and gamma aminobutyric acid (GABA) transport 27, and cell maturation 28 and morphology 29. These TrkB-FL-independent actions might rely on interactions established by a highly conserved and isoform-specific TrkB-T1 C-ter sequence (FVLFHKIPLDG) 13,23 although the only TrkB-T1-interacting protein previously identified is RhoGDP dissociation inhibitor 1 (RhoGDI1), found in astrocytes 29. This protein controls the family of Rho GTPases, regulatory proteins that link surface receptors to cytoskeleton organization, regulation of gene transcription and neuronal survival/death 30.

TrkB-T1 expression is upregulated in multiple disorders, including stroke, spinal cord injury, several neurodegenerative diseases (NDDs) or Down syndrome 31. In stroke, while TrkB-FL decreases in the infarct core, TrkB-T1 levels increase in the surrounding astrocytes 32, promoting ischemic damage and contributing to oedema formation 33. This TrkB imbalance could be reproduced in a model of *in vitro* excitotoxicity, where it significantly contributed to neuronal death 9. This is important because excitotoxicity is not only a mechanism central to stroke but it is also associated to other acute (hypoglycemia, acute trauma) or chronic CNS disorders such as NDDs 34. Three excitotoxicity-induced mechanisms contribute to aberrant TrkB function: (1) modification of normal ratio of isoform mRNAs, favoring TrkB-T1 mRNA 9,35; (2) TrkB-FL calpain cleavage, resulting in a non-functional truncated receptor similar to TrkB-T1 and a cytosolic TK-containing fragment 36; (3) regulated intermembrane proteolysis (RIP) of both isoforms by sequential action of metalloproteinases (MPs), shedding identical ectodomains acting as BDNF scavengers, and γ-secretases, releasing the isoforms intracellular domains (ICDs) 36. RIP is a major mechanism of TrkB-T1 regulation both *in vitro* and after ischemia while it is only secondary for TrkB-FL 36. Similarly to other RIP substrates, TrkB-T1-ICD could trigger local signaling or modify gene expression after nuclear translocation 37. Altogether, these pathological mechanisms lead to abnormal BDNF/TrkB-signaling which contributes to excitotoxicity, pointing to TrkB-FL and TrkB-T1 as relevant therapeutic targets for treatment of stroke and excitotoxicity-associated pathologies. We have previously designed a cell-penetrating peptide (CPP) able to cross the BBB and plasma membrane that interferes excitotoxicity-induced TrkB-FL retrograde transport to the Golgi complex, secondarily preventing receptor processing 38 and organelle disruption 39, a neurodegeneration hallmark. In a model of ischemia, this neuroprotective peptide efficiently reduces infarct size and neurological damage 38. The significant role of TrkB-T1 in excitotoxicity, coupled with the potential to target both neurons and glial cells, strongly justifies the development of alternative or complementary therapeutic strategies focused on this isoform. In order to do that, we relied again on CPPs since they have important advantages for non-invasive delivery of therapeutic compounds to the CNS: translocation capacity across cell membranes, ability to infiltrate many different cell types, large cargo capacity and low toxicity 40,41. Although the pharmacokinetic properties of CPPs present some limitations, promising results have been recently obtained in phase 3 clinical trials for acute ischemic stroke 42,43.

Here, we hypothesize that isoform-specific TrkB-T1 interactions might play a relevant role in neurotoxicity and/or reactive gliosis. Therefore, their interference might prevent those pathological processes and decrease the ischemic damage. We have designed TrkB-T1-derived CPPs to isolate and identify the isoform interactome in basal and excitotoxic conditions. We also demonstrate that these peptides regulate neuroinflammation and are neuroprotective against *in vitro* or *in vivo* excitotoxicity, suggesting that they might be relevant for therapy of human stroke and other pathologies associated to excitotoxicity and neuroinflammation.

## Methods

Reagents and resources are described in the Table S1.

### Experimental models

Animal procedures were performed following European Union Directive 2010/63/ EU and approved by ethics committees from CSIC and Comunidad de Madrid (Ref PROEX 276.6/20).

#### Mice model of ischemia by photothrombosis

Permanent focal ischemia was induced in cerebral cortex of adult male and female mice by microvascular photothrombosis as described 38 with modifications. Brain injury involves vascular endothelium damage and platelet activation, followed by microvascular thrombotic occlusion of a particular region 44 selected for illumination using a stereotaxic frame. Coordinates were +0.2 AP, +2 ML relative to Bregma and damaged area corresponded to primary motor and somatosensory cortex. For neuroprotection, a single dose (3 nmol/g) of peptides TMyc or TT1_Ct_ (see below) was administered 1 h after damage initiation. Further details of the procedures, including measurement of infarct volume and evaluation of motor coordination and balance, are detailed as Supplementary material.

#### Primary cultures of rat cortical neurons and treatment

Cultures were prepared from cerebral cortex of 18-day-old Wistar rat embryos, both genders indistinctly included 45. Unless otherwise indicated, glial growth was inhibited after 7 days *in vitro* (DIVs), experimental treatments taking place after 12 DIVs. These cultures present a high percentage of neurons, combined with glial cells, mostly astrocytes 9. To induce excitotoxicity, cultures were incubated with NMDA (100 µM) and its co-agonist glycine (10 µM), herein denoted simply as NMDA, which induce a strong excitotoxic response in mature neurons but have no effect on astrocyte viability 46,47. When indicated, cultures were preincubated for 30 min with indicated concentrations of Tat-derived CPPs before NMDA treatment, peptides being kept in the medium along treatment. Total cell or neuronal viability assessment was established by the thiazolyl blue formazan (MTT) reduction assay. Further details are provided as Supplementary material.

### Pull down assays and proteomic analysis

Cultures incubated with biotinylated peptides and treated as indicated were lysed at 4°C in NP-40 lysis buffer. Equal protein amounts were combined with streptavidin-agarose beads and incubated for 1.5 h for specifically-bounded protein isolation. Peptides and their interacting proteins were released by incubation at 50°C for 40 min in RIPA modified buffer. Peptide-interacting proteins were collected and subjected to LC-ESI-MS/MS (HR, medium gradient) protein identification using a mass spectrometer (Orbitrap Exploris 240) and Proteome Discoverer software for analysis (CNB Proteomics Facility, CSIC). Further procedure details are provided as Supplementary material.

### Cell transfection and gene reporter assays

Plasmids contained minimal CREB or MEF2 response elements upstream firefly luciferase reporter gene (respectively, pCRE or pMEF2; see details in Table S1). Primary cultures were transfected as detailed in the Supplementary material and incubation proceeded to complete 24 h. For quantitation, 5-7 totally independent experiments were repeated as indicated, each one including technical quadruplicates for every condition.

### RNA extraction and qPCR assay

Total RNA was extracted and analyzed as detailed in the Supplementary material. For each independent experiment, we analyzed technical triplicates for every sample and performed a total of 5 completely independent experiments. Data were normalized, for each experiment, with housekeeping genes: neuronal specific enolase (NSE, for genes having neuronal expression) or glyceraldehyde-3-phosphate dehydrogenase (GAPDH, for GFAP, only expressed in glial cells).

### Peptide visualization and immunocytochemistry in cultures

Cells grown on coverslips were incubated for 1 h with biotin-conjugated peptides Bio-TMyc or Bio-sTT1_Ct_ (25 µM) or left untreated, and then fixed for 30 min with 4% paraformaldehyde in PBS. After blocking and permeabilization, incubation with Fluorescein Avidin D and immunocytochemistry were as described in the Supplementary material.

### Peptide visualization and immunohistochemistry in brain cortex

Vehicle (saline), Bio-TMyc or Bio-TT1_Ct_ (3 nmol/g) were retro-orbitally injected in mice one hour after induction of photothrombotic damage or, alternatively, undamaged animals. In the first case, animals were sacrificed 5 h after damage induction while, the second group was sacrificed 30 min after peptide administration. The details of brain fixation and cryoprotection for peptide visualization and/or immunohistochemistry are provided in the Supplementary material.

### Statistical analysis

Except for box and whiskers plots, data are expressed as mean ± standard error of the mean (SEM) of at least four independent experiments. The details of the number of completely independent experiments done (n) and the specific statistical test applied can be found in each respective figure legend. For viability, mRNA and gene reporter assays, technical replicates were included in each independent experiment. Treatment assignation was performed at random.

Statistical analysis was performed in GraphPad Prism 8.0.2. The normality of the data was analyzed by Saphiro-Wilk test. In all cases *P*-value significance is considered as: *P < 0.05, ** P < 0.01, *** P < 0.001, **** P < 0.0001. A *P*-value larger than 0.05 is considered non-significant (n.s.).

For the *in vivo* neuroprotection study, the number of required animals was calculated using G*Power 3.1.9.7 software, with a statistical power of 0.8 and an alpha error probability of 0.05.

## Results

### Identification and targeting of TrkB-T1-specific interactome with a CPP containing the isoform C-ter

**Figure 1:**
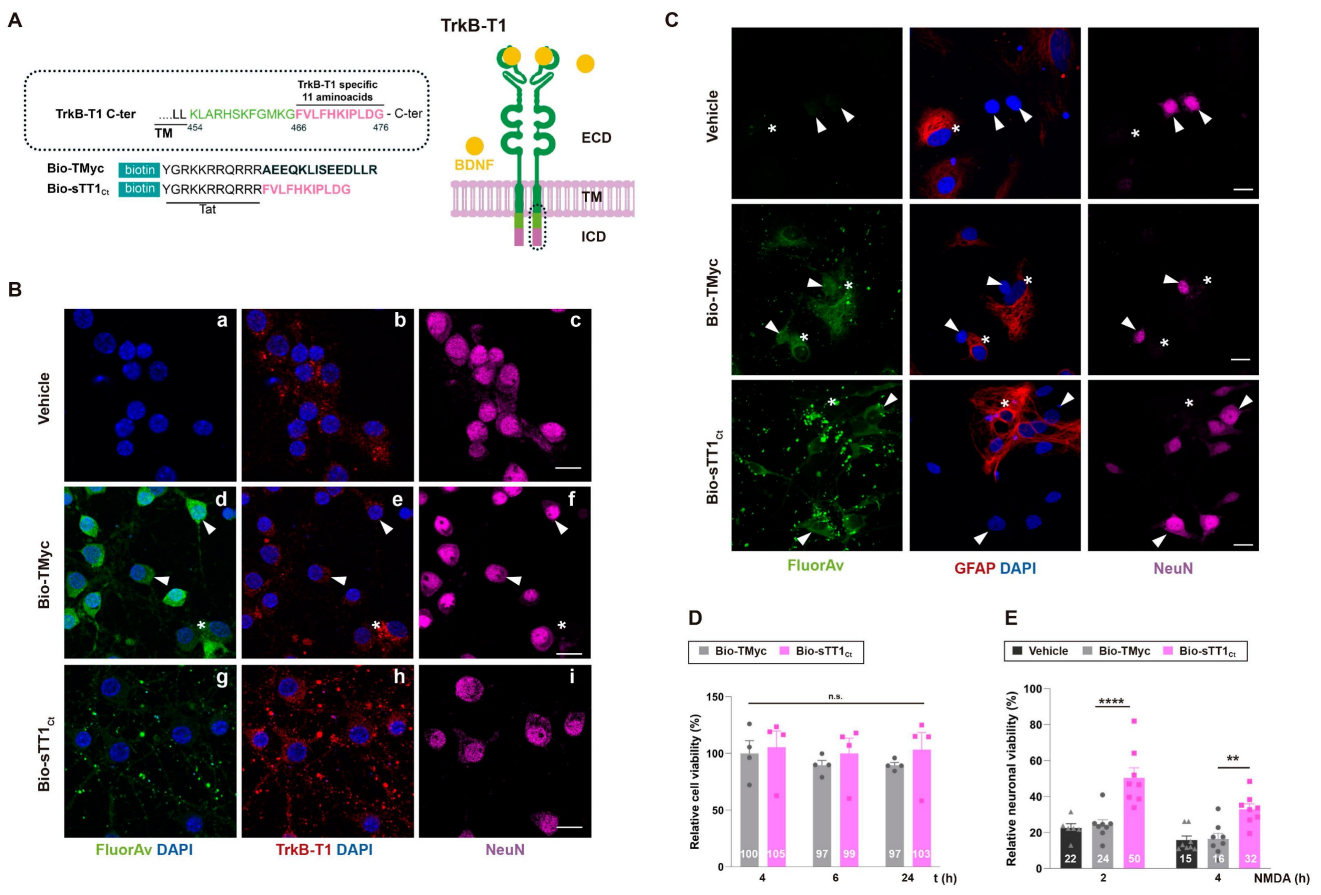
** Validation of isoform-specific CPPs as tools for identification of TrkB-T1 interactome and prevention of neuronal death by excitotoxicity.** (A) Structure of TrkB-T1 receptor, indicating the extracellular domain (ECD), responsible of brain derived neurotrophic factor (BDNF)-binding, the transmembrane segment (TM) and the short intracellular domain (ICD). The precise sequence corresponding to the TrkB-T1 C-ter (dotted oval) is indicated. It contains a region shared with TrkB-FL (black and green) followed by the TrkB-T1-specific sequence (pink). Biotin (Bio)-labelled Tat-derived CPPs, containing this isoform-specific sequence (Bio-sTT1_Ct_) or unrelated sequences for the control peptide (Bio-TMyc), are also indicated. (B) Immunocytochemistry assays of primary cortical cultures treated with Bio-sTT1_Ct_, Bio-TMyc (25 µM) or vehicle for 30 min. TrkB-T1 and Bio-sTT1_Ct_ distribution were analyzed with an isoform specific antibody (red). Peptide visualization with Fluorescein Avidin D (green) shows that Bio-sTT1_Ct_ presents a pattern similar to that observed for endogenous TrkB-T1. Bio-TMyc distribution was visualized in Neu+ (arrowheads) and Neu- (asterisk), corresponding respectively to neurons and presumably astrocytes (d-f). Scale bar, 10 µm. (C) Analysis of cultures treated as before with specific antibodies for astrocytes (GFAP, red) or neurons (NeuN, magenta). Peptide visualization with Fluorescein Avidin D (green) shows distribution in both Neu+ neurons (arrowheads) and GFAP+ astrocytes (asterisk). Scale bar, 10 μm. (D) Cell viability of cortical cultures treated with Bio-sTT1_Ct_ and Bio-TMyc (25 µM) for 4, 6 or 24 h. Means ± SEM and individual points are presented relative to values obtained for 4 h of Bio-TMyc treatment (100%). Data were analyzed using two-way ANOVA test followed by *post hoc* Bonferroni test, *n* = 4. (E) Neuronal viability in cultures incubated with Bio-TMyc or Bio-sTT1_Ct_ (25 µM) for 30 min and treated with NMDA for 2 or 4 h. Means ± SEM and individual points are presented relative to the values obtained for untreated cells (100%). Data were analyzed using two-way ANOVA test followed by *post hoc* Bonferroni test, *n* = 8.

TrkB-T1-independent functions might require particular protein interactions established by its highly conserved and isoform-specific C-ter (aa 466-476, Figure 1A) 48, which could be altered by specific biological conditions. This is the case of RhoGDI1, the only TrkB-T1-interacting protein previously identified, released from astrocyte constitutive complexes by BDNF/TrkB-T1-binding 29. Additional TrkB-T1-specific interactions established in neurons and/or astrocytes and how they might be affected by BDNF-binding or excitotoxicity are presently unknown. Thus, we designed CPP Bio-sTT1_Ct_ containing three elements: 1) a biotin molecule that allows peptide visualization and pull-down of interacting proteins; 2) a HIV-1 Tat short basic domain which allows crossing of BBB and plasma membrane to attached cargoes 49,50 and 3) the isoform-specific sequence (Figure 1A). As a negative control, we used a similar peptide containing unrelated sequences corresponding to c-Myc (Bio-TMyc), which could enter NeuN+ (arrowheads) and NeuN- cells (asterisk) present in mixed primary cultures of rat embryonic cortex (Figure 1Bd-f) but had no effects on neuronal viability in basal or excitotoxic conditions 38,45. The major cell-subtype in these cultures corresponds to Neu+ neurons which seem to express lower TrkB-T1 levels compared to NeuN- cells also present in the cultures (Figure 1Be), presumably astrocytes. In fact, primary cultures of cortical astrocytes expressed 7-fold higher TrkB-T1 levels than those found in the mixed cultures of neurons and astrocytes employed here (Figure S1). A vesiculated Bio-sTT1_Ct_ distribution was observed in neurons (Figure 1Bg), resembling that of endogenous TrkB-T1 (Figure 1Bb, e). In fact, staining with Fluorescein Avidin D (Figure 1Bg) and the isoform-specific antibody, prepared with the same TrkB-T1 sequence included in Bio-sTT1_Ct_ (Figure 1Bh), suggested that Bio-sTT1_Ct_ and TrkB-T1 mostly colocalized. A CPP subcellular distribution guided by the specific protein sequences contained might explain the differences observed between Bio-MTMyc (Figure 1Bd) and Bio-sTT1_Ct_ (Figure 1Bg) patterns. Next, we confirmed peptide entry into neurons and astrocytes, as suggested by previous experiment, by double staining with cell-type specific markers, respectively NeuN and GFAP (Figure 1C). Bio-sTT1_Ct_ and Bio-TMyc were found inside both NeuN+ (arrowheads) and GFAP+ (asterisks) cells present in the mixed cultures, corresponding respectively to neurons and astrocytes.

Above data suggested that Bio-sTT1_Ct_ might interfere protein interactions established by TrkB-T1 C-ter required, for example, for neuronal death promotion in a context of excitotoxicity. Accordingly, cell viability was not affected by Bio-TMyc and Bio-sTT1_Ct_ in basal conditions (Figure 1D) while they differently affected the strong and time-dependent decrease in neuronal viability induced by NMDAR overactivation with co-agonists NMDA (100 µM) and glycine (10 µM), herein simply denoted as NMDA (Figure 1E). Preincubation with Bio-sTT1_Ct_ had a significant neuroprotective effect after 2 (50 ± 5%) or 4 h (32 ± 3%) of excitotoxicity compared to Bio-TMyc-treated cultures (respectively, 24 ± 3%, ****P < 0.0001 and 16 ± 3%, **P < 0.01; *n* = 8).

**Figure 2:**
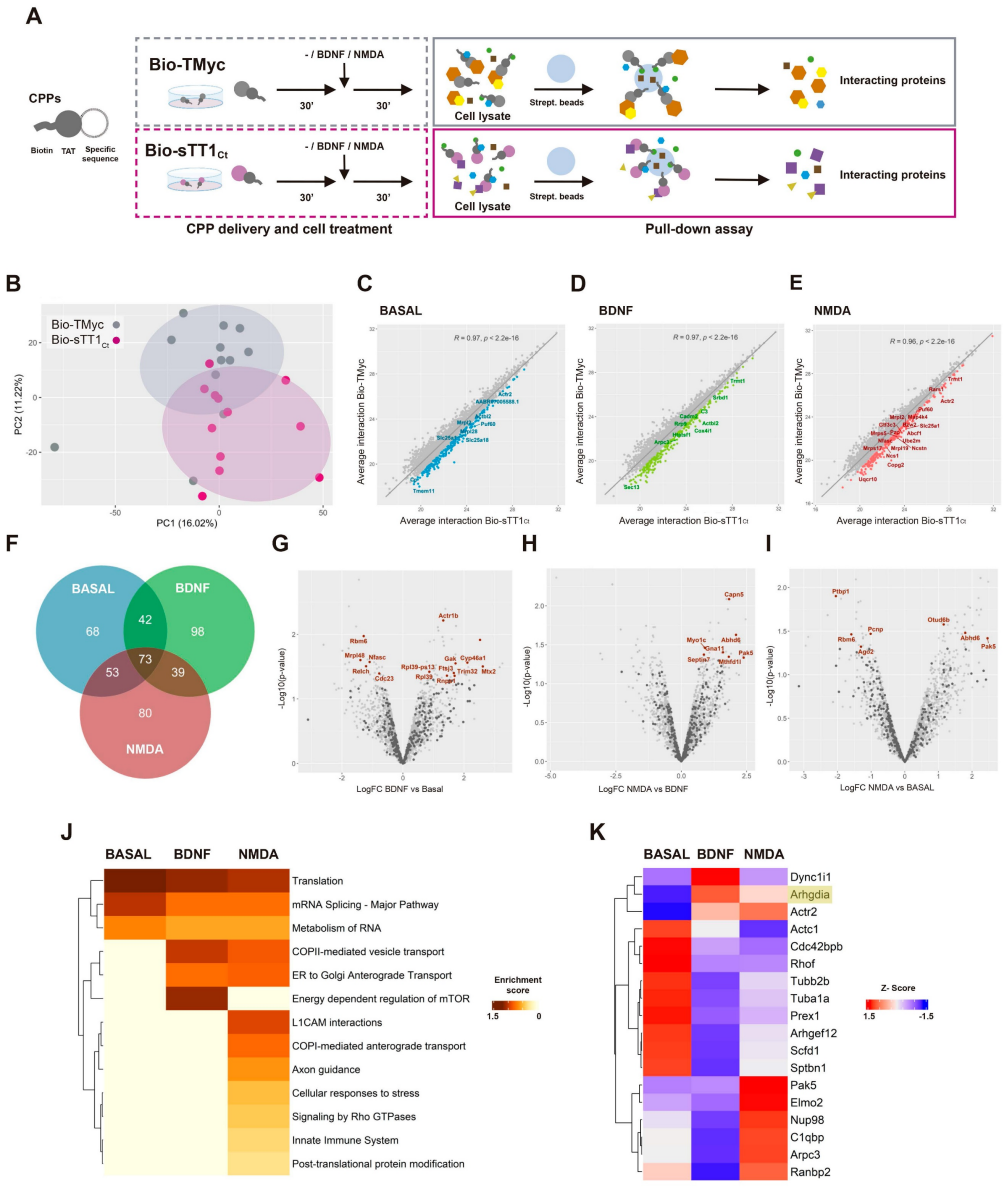
** Bio-sTT1_Ct_ as a tool to approach TrkB-T1 specific interactome in different biological conditions.** (A) Experimental design of pull-down assays to isolate Bio-TMyc and Bio-sTT1_Ct_ interacting proteins. Cultures were incubated with Bio-sTT1_Ct_ or Bio-TMyc (25 µM) for 30 min before treatment with BDNF (100 ng/ml) or NMDA for 30 min. After that, cell lysates were combined with streptavidin agarose beads to isolate the CPP-interacting proteins. (B) Principal Component Analysis of Bio-TMyc and Bio-sTT1_Ct_ pull-down isolates. Samples are represented using the first (PC1) and second (PC2) components of the analysis. (C-E) Pearson's correlation of the average protein interactions established by Bio-TMyc and Bio-sTT1_Ct_ in basal conditions (C), or after BDNF (D) or NMDA treatment (E). Colored points represent the top 20% proteins whose residuals are furthest from the correlation line and have higher levels of binding to Bio-sTT1_Ct_. (F) Venn diagram representing the number of proteins selected for each condition following the criteria described above. (G-H) Volcano plot presenting the results of the differential analysis of interacting proteins comparing BDNF vs basal conditions (G), NMDA vs BDNF (H) and NMDA vs basal conditions (I). Log_2_FC and -Log_10_(p-value) are shown. Proteins selected with the criteria explained above are represented as dark grey points while proteins showing statistically significant differences are labelled and presented as brown dots (p-value < 0.05). FDR = 9.52% (G), 4.15% (H) or 9.55% (I). FC, fold change. (I) Pathway enrichment analysis of selected proteins for basal, BDNF and NMDA conditions. A heatmap showing the enrichment score for each pathway (Reactome.db) at the different conditions is presented. (J) Comparison of RhoGDI (*Arhgdia*) (highlighted) and our selected proteins annotated in "Signaling by Rho GTPases" Reactome pathway in basal, BDNF and NMDA conditions. A heatmap showing the z-score indicating levels for each protein at the different conditions is presented.

These results are suggestive of Bio-sTT1_Ct_ being able to interfere protein interactions established by TrkB-T1 inside cells and, thus, this peptide could be used to isolate and identify the isoform specific interactome in different biological conditions. Cultures were incubated with Bio-TMyc or Bio-sTT1_Ct_ (30 min) before treatment with BDNF (100 ng/ml) or NMDA (100 µM) for 30 additional minutes, or left untreated (Figure 2A). Intracellular complexes formed by biotin-labelled peptides were isolated from lysates using streptavidin-agarose precipitation. Then, proteins interacting with Bio-TMyc and Bio-sTT1_Ct_ were identified by a label free proteomic assay, obtaining a total of 4697 proteins for the three different biological conditions and four independent experiments analyzed. Some proteins might be interacting with shared peptide elements such as the biotin moiety or the Tat sequence, or even streptavidin-agarose (Figure 2A). Therefore, we evaluated by a Principal Component Analysis (PCA) whether the interactome profiles obtained were different for both peptides (Figure 2B). The first and second principal components of this analysis (respectively, PC1 and PC2) split the samples into two groups, proving a differential profile of interactions for Bio-TMyc and Bio-sTT1_Ct_.

To emphasize those proteins more specifically interacting with Bio-sTT1_Ct_, we calculated Pearson's correlation between average protein interactions established by Bio-TMyc and Bio-sTT1_Ct_ at the different conditions (Figure 2C-D). Then, we analyzed the correlation line residuals and selected the top 20% of proteins having higher average interaction with Bio-sTT1_Ct_ compared to Bio-TMyc (highlighted as colored dots). Some of the 453 selected proteins maintained Bio-sTT1_Ct_-interaction at all three conditions while others were affected by BDNF or NMDA treatment (Figure 2F). Interestingly, gene ontology (GO) analysis of biological process enrichment (string.db) associated with Bio-sTT1_Ct_-interacting proteins presented terms related to gene expression and mRNA regulation, including splicing, at all treatment conditions (Figure S2). Only upon NMDA treatment, other biological process terms appeared such as cytoskeletal reorganization and cell division (Figure S2C).

Then, we further characterized the impact of cell conditions on Bio-sTT1_Ct_ interactions by differential analysis of binding for previously selected proteins (Figure 2G-I). Significant differences were found in all comparisons (named proteins in corresponding figures, summarized in Table S2), proving that some Bio-sTT1_Ct_-protein complexes were affected by specific stimuli. Thus, BDNF induced Bio-sTT1_Ct_-binding of proteins involved in endocytosis and molecular trafficking (*Gak*), cholesterol metabolism (*CYP46A1*), pre-rRNA processing (*Ftsj3*) or mitochondria-endoplasmic reticulum contact sites (*MERCS*) (*Mtx2*/*Grp75*; Figure 2G). Meanwhile, excitotoxicity promoted significant changes in binding of proteins involved, among others, in gene expression (*Otud6b*, *Ago2*, *Rbm6* and *Ptbp1*; Figure 2I) and cytoskeletal remodeling (*Septin7*, *Pak5*, *Capn5*; Figure 2H-I). Furthermore, comparison of enriched pathways (Reactome, string.db) among tested conditions showed general Bio-sTT1_Ct_-interaction with proteins involved in translation, RNA metabolism and splicing, as seen before in the biological process terms (Figure S2), while BDNF treatment enriched pathways related to vesicle transport and mTOR regulation (Figure 2J). In fact, vesicle transport was also increased with NMDA, while other pathways key for neurotoxicity promotion such as innate immune system, L1CAM interactions, cellular response to stress, axon guidance and RhoGTPases activity specifically appeared. These changes suggest an important role of TrkB-T1 C-ter in excitotoxicity development by interaction with proteins involved in this pathological process.

Next, we analyzed in detail the group of Bio-sTT1_Ct_-interacting proteins included in Reactome function "Signaling by RhoGTPases" (Table S3 and Figure 2K), a pathway enriched after NMDA treatment (Figure 2J). RhoGDI (*Arhgdia* encoded), which is expressed in neuronal-enriched cortical cultures (Figure S3), presented a very low binding to Bio-sTT1_Ct_ and did not pass the established selection criteria, being included in the analysis only as a reference (Figure 2K). This result suggests that TrkB-T1/RhoGDI interaction might be different in neurons and astrocytes 29. Regarding other RhoGTPase-related proteins (Table S3), most of them presented a very reduced binding after BDNF stimulation while two opposite Bio-sTT1_Ct_-interacting patterns were found in excitotoxicity compared to basal conditions. The interaction was downregulated by NMDA for an important group of proteins, including cytoskeleton components (*Actc1, Tubb2b, Tuba1a, Sptbn1*), Rho and Rac GEFs (respectively *Arhgef12* and *Prex1*) or a Cdc42 effector (*Cdc42bpb*). In contrast, Bio-sTT1_Ct_-interaction was promoted by NMDA for some proteins including *Actr2* and *Arpc3*, components of the Arp2/3 complex mediating actin polymerization, *C1qbp*, involved in inflammation, *Nup98* and *Ranbp2*, components of the nuclear pore complex (NPC) and involved in RNA transport, *Elmo2*, a regulator of Rac GTPase activity and *Pak5*, a downstream effector of Rac1 and Cdc42 GTPases.

Altogether, these results represent a first comprehensive analysis of isoform-specific TrkB-T1 interactions and their modulation in cell models of pathophysiology. Some of the identified TrkB-T1-interacting proteins might contribute to the isoform central role in excitotoxicity and ischemia and help to explain preliminary results showing a neuroprotective effect of Bio-sTT1_Ct_ (Figure 1E), unveiling a new therapeutic strategy to treat stroke and additional excitotoxicity-associated pathologies that merits further investigation.

### Design of peptide TT1_Ct_ and mechanism of neuroprotection in cellular models of excitotoxicity

**Figure 3:**
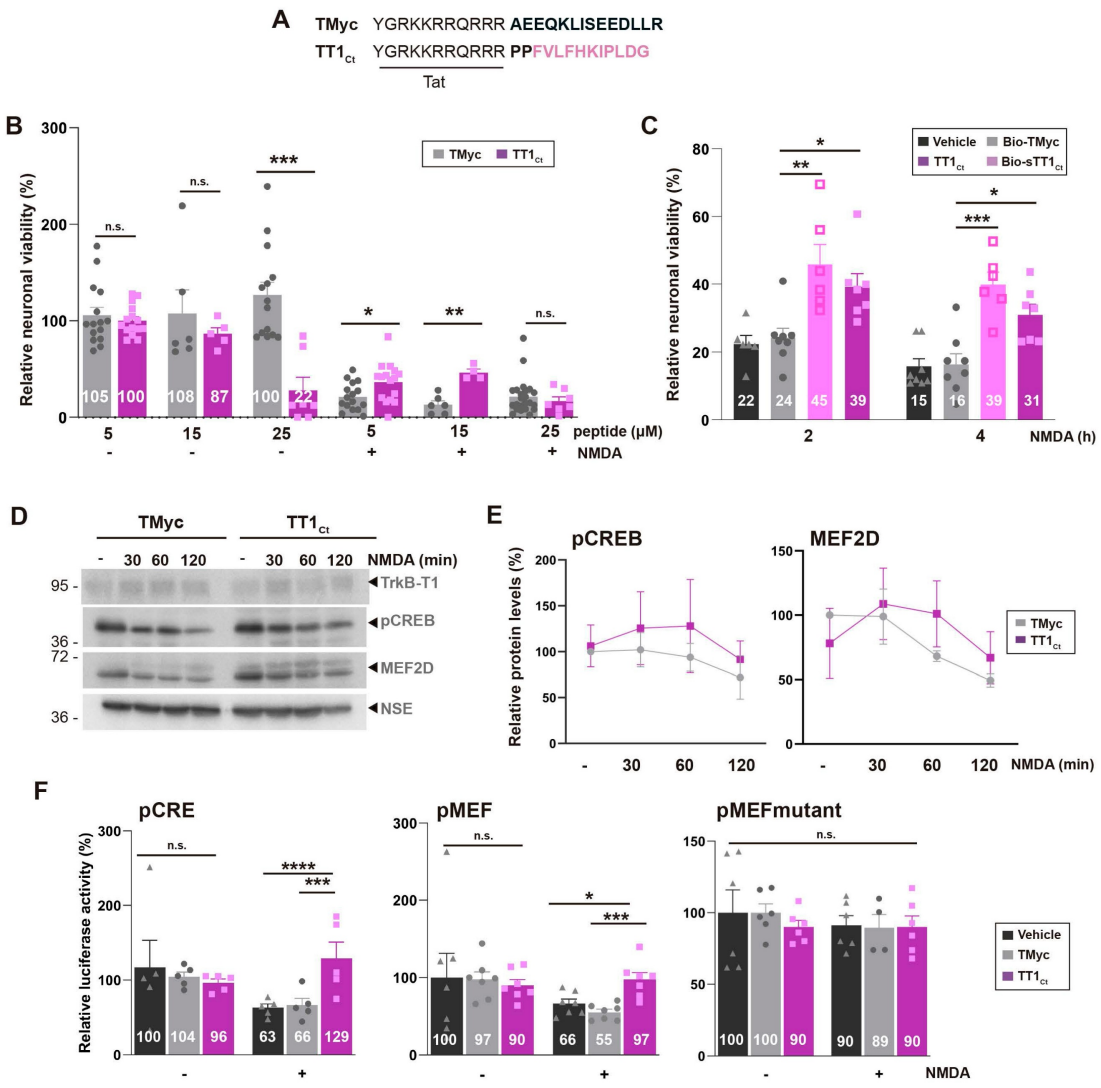
** TrkB-T1-derived peptide TT1_Ct_ is neuroprotective against *in vitro* excitotoxicity and prevents a decrease in CRE and MEF promoter activities induced by the excitotoxic injury.** (A) Sequence of control peptide (TMyc) and TrkB-T1-derived CPP (TT1_Ct_). (B) Neuronal viability in cultures incubated with TMyc or TT1_Ct_ (5, 15 or 25 µM) for 30 min and then treated with NMDA (100 µM) for 2 h or left untreated. Individual data and means ± SEM are presented relative to values obtained in the untreated cells (100%). Data were analyzed using Kruskal-Wallis test followed by Mann-Whitney U-test, *n* = 6-16. (C) Neuronal viability in cultures incubated with Bio-TMyc, Bio-sTT1_Ct_ or TT1_Ct_ (15 µM) for 30 min and then treated with NMDA (100 µM) for 2 or 4 h. Individual data and means ± SEM are presented relative to values obtained for untreated cells (100%). Data were analyzed using two-way ANOVA test followed by *post hoc* Bonferroni test, *n* = 6-8. (D) Western Blot analysis of TrkB-T1, pCREB and MEF2D levels in cultures incubated with TMyc or TT1_Ct_ (15 µM) for 30 min followed by treatment for 30, 60 or 120 min with NMDA. A representative experiment is shown. (E) Quantitation by densitometric analysis of pCREB and MEF2D levels. Means ± SEM are presented relative to basal conditions in the presence of TMyc (100%), *n* = 5. (F) Effect of excitotoxicity and TT1_Ct_ treatment on CRE and MEF2 promoter activity. Cultures transfected with plasmids containing minimal CREB or MEF2 response elements (respectively, pCRE and pMEF) or pMEFmut were preincubated with peptides as above and treated with NMDA for 2 h or left untreated. Individual results and means ± SEM are presented relative to luciferase expression in untreated cultures. Data was analyzed by two-way ANOVA test followed by *post hoc* Bonferroni test, *n* = 5-7. For cultures transfected with pCRE and pMEF, treated with vehicle or TMyc, differences between -/+ NMDA are statistically significant although not shown for simplicity.

To further characterize the effect of TrkB-T1 interference in excitotoxicity, we designed a new CPP, TT1_Ct_ (Figure 3A). This peptide lacks the biotin molecule, to avoid possible unwanted effects, and contains two spacer prolines separating Tat and isoform-specific sequences, to disrupt a possible regular secondary structure between these functionally-independent moieties that might affect peptide stability and efficacy. As a negative control, we used TMyc, a similar peptide having unrelated c-Myc sequences (Figure 3A) 38,45. We first analyzed TT1_Ct_ effects on neuronal viability under basal conditions or *in vitro* excitotoxicity (Figure 3B). In a dose-response experiment, TT1_Ct_ resulted toxic for non-stimulated cultures at the higher dose employed (25 µM), probably mimicking some TrkB-T1-ICD neurotoxic effects. In contrast, TT1_Ct_ was not intrinsically toxic at concentrations lower than 25 µM while it was strongly neuroprotective against excitotoxicity. Thus, neuronal viability was 46 ± 7% in cultures preincubated with TT1_Ct_ (15 µM, 30 min) before NMDA treatment (2 h), significantly higher values compared to TMyc-preincubated cells (13 ± 9%, **P < 0.01; *n* = 8). Next, in a time-course experiment, we compared the neuroprotective efficacy of TrkB-T1-derived peptides, Bio-sTT1_Ct_ and TT1_Ct_ (15 µM) (Figure 3C), finding similar neuroprotective effects. Neuroprotection due to TT1_Ct_-preincubation was maintained when the peptide was applied at the time of damage induction but lost if added at later times (Figure S4), suggesting that this peptide affects processes taking place in cultures at early times of excitotoxicity.

Our next goal was to investigate the mechanism of TT1_Ct_ neuroprotection. We have previously shown that excitotoxicity induces a progressive increase in TrkB-T1 levels 9 which probably contributes to neuronal death. Other proteins also affected by NMDAR overactivation are TFs CREB and MEF2. Levels of S133 phosphorylated CREB (pCREB), generally considered the active protein, are strongly reduced early upon excitotoxicity induction probably due to phosphatase activation. The result is CREB shut-off, inhibition of BDNF expression and death of mature neurons 51. In addition, there is a severe MEF2D decrease in cultures subjected to excitotoxicity 38, probably due to caspases or calpain action 52,53. The effect of excitotoxicity in cultures incubated with TMyc (15 µM, 30 min) before NMDA treatment (0-120 min) was as expected (Figure 3D-E). At these early times of excitotoxicity, TT1_Ct_ did not have significant effects in TrkB-T1 regulation (Figure 3D) but partially preserved pCREB and MEF2D, although differences were not statistically significant compared to TMyc-treated cultures (Figure 3E). Neuron-specific enolase (NSE), a protein not affected by NMDA, was used as a loading control and for protein normalization. Altogether, these results suggest that one mechanism of TT1_Ct_ neuroprotection might be mediated by CREB and MEF2D through gene expression regulation.

To further explore the potential role of TT1_Ct_ on CREB and MEF2, we performed reporter assays using promoters with minimal response elements (respectively, pMEF2 54 or pCRE 21; see Table S1) regulating luciferase expression. In basal conditions, TT1_Ct_ and TMyc (15 µM) had no significant effects on pCRE and pMEF promoter activities compared to untreated cultures (Figure 3F). Excitotoxicity provoked a dramatic decrease in luciferase activity in vehicle or TMyc-preincubated cultures which was significantly prevented by TT1_Ct_. An inactive pMEF mutant with reduced luciferase expression was unresponsive to NMDA or peptide treatment, proving that excitotoxicity regulates MEF2-promoter activity and TT1_Ct_ has a specific effect on it.

**Figure 4:**
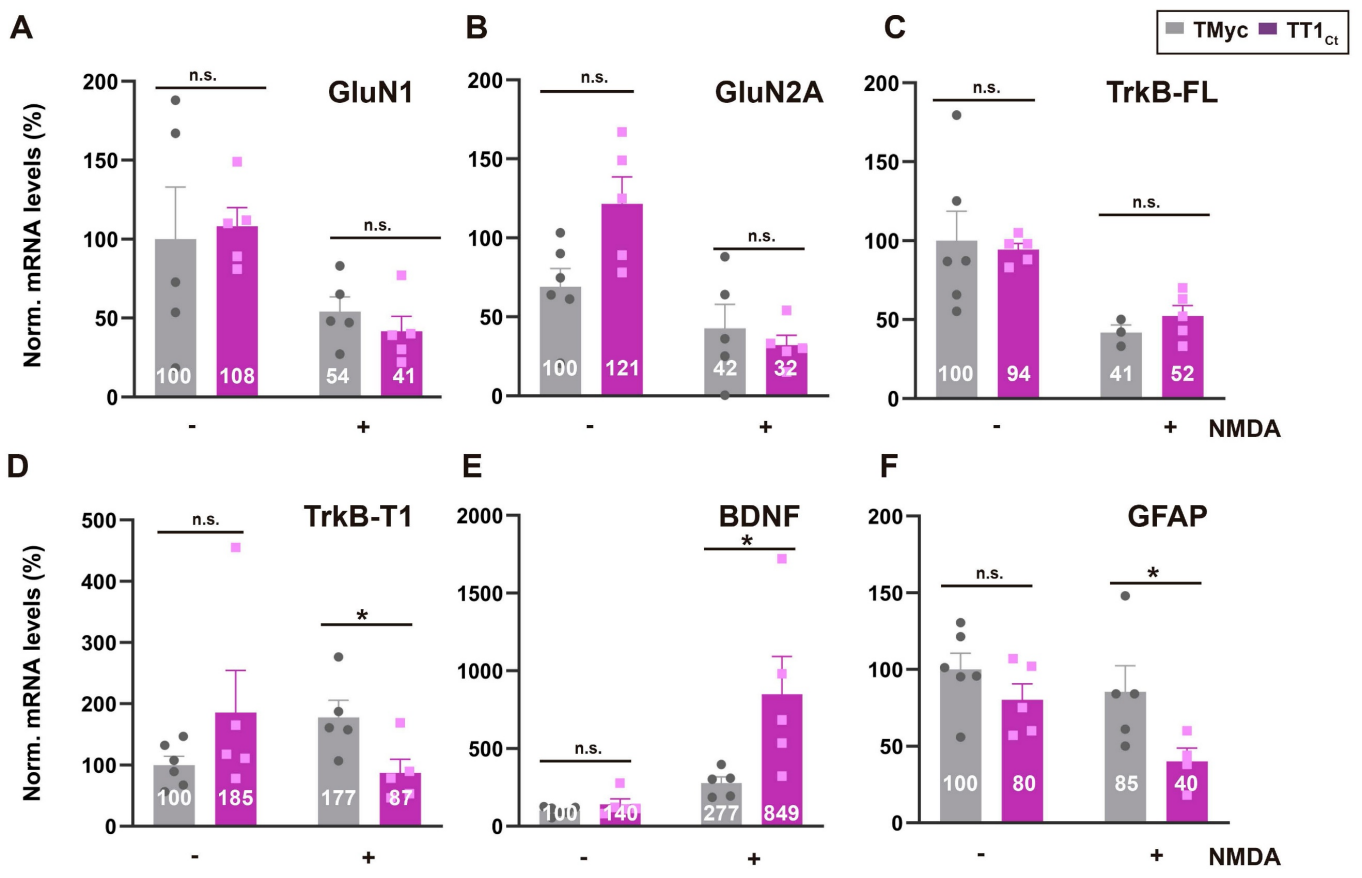
** TT1_Ct_ interferes transcriptional changes induced by excitotoxicity affecting expression of genes involved in survival/death choices.** Cultures preincubated with TT1_Ct_ or TMyc (15 µM) for 30 min were treated with NMDA for 4 h or left untreated. Levels of mRNAs encoding for NMDAR-subunits GluN1 (A) and GluN2A (B) or TrkB-FL (C) and TrkB-T1 isoforms (D) were normalized to those of NSE. Levels of BDNF (E) and GFAP mRNA (F) were normalized relative to GAPDH. Individual values and means ± SEM are presented relative to gene expression in cultures treated with TMyc (100%). Data were analyzed by two-way ANOVA test followed by *post hoc* Tukey's or Kruskal Wallis test followed by *post hoc* Dunn's test, *n* = 5.

Above results are important because these TFs are central to neuronal survival induced by synaptic activity 55,56 or neurotrophins 15,16. In addition, CREB also regulates the activity of astrocytes in response to neurotransmitters 57-59. To establish if TT1_Ct_ alters excitotoxicity-induced transcriptional changes in neurons and/or astrocytes, we next analyzed levels of mRNAs encoding proteins involved in survival/death choices (Figure 4). As expected, NMDA induced a strong decrease in levels of mRNAs encoding GluN1 60, GluN2A 60 and TrkB-FL 9 in TMyc or TT1_Ct_ presence (Figure 4A-C). In contrast, we confirmed a NMDA-induced increase in TrkB-T1 mRNA 60 in TMyc-treated cells, that was counteracted by TT1_Ct_ action (Figure 4D). For BDNF, excitotoxicity showed a tendency to increase mRNA levels in TMyc presence, in agreement to previous results 61, and such accumulation was strongly exacerbated by TT1_Ct_ (Figure 4E). Finally, this peptide decreased GFAP mRNA levels relative to those found in TMyc-treated cultures in excitotoxicity (Figure 4F). In conclusion, TT1_Ct_ does not seem to affect the transcriptional downregulation of the analyzed neuronal genes induced by excitotoxicity while it strongly modifies transcription of genes expressed in astrocytes (GFAP) or both neurons and astrocytes (TrkB-T1 and BDNF), where it counteracts excitotoxic effects. Altogether, we conclude that interference of TrkB-T1 interactions by TT1_Ct_ during excitotoxicity maintains levels and promoter activities of CREB and MEF2, which regulate the transcription of TrkB-T1 itself and, other target genes critical to neuronal survival and astrocyte activation.

### Effects of TT1_Ct_ in a model of ischemic stroke induced by photothrombosis

**Figure 5:**
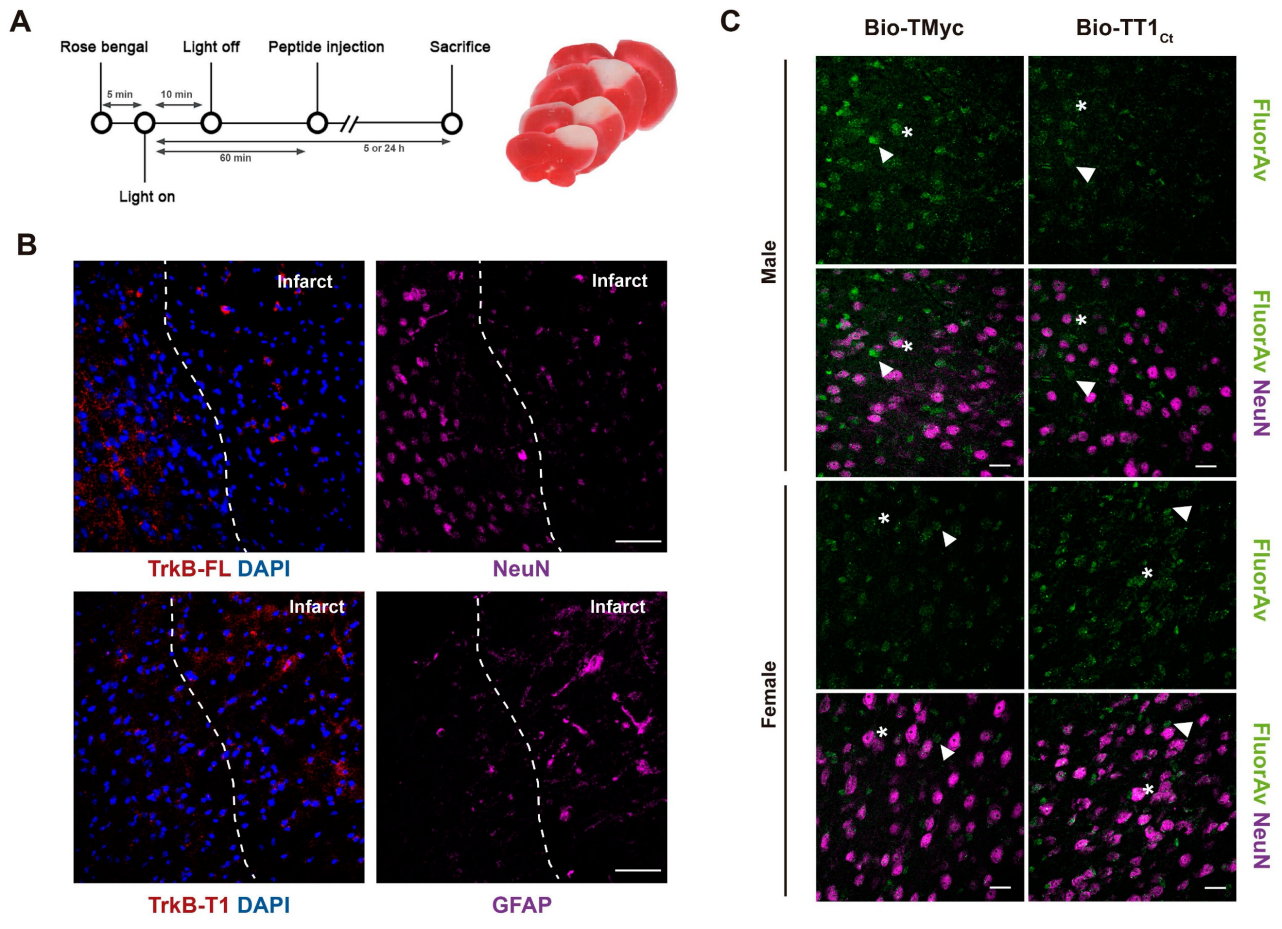
** TT1_Ct_ brain distribution in a mouse model of permanent ischemia showing upregulation of TrkB-T1+/GFAP+ cells in the interface between ischemic and non-ischemic tissue.** (A) Experimental design to analyze *in vivo* effects of TMyc and TT1_Ct_. Permanent vessel occlusion and focal brain damage were induced in mice by cold-light irradiation after Rose Bengal i.v. injection. CPPs (3 nmol/g) were i.v. injected 1 h after damage initiation. Animals were sacrificed 5 or 24 h after injury onset as indicated. Representative 1 mm brain coronal slices stained with TTC after 24 h of insult are shown. (B) Immunohistochemistry of brain coronal sections prepared from TMyc-treated male animals 5 h after insult stained with isoform-specific TrkB antibodies (TrkB-FL and TrkB-T1), NeuN, GFAP and DAPI. Maximal projection of representative confocal microscopy images showing cortical areas of the infarct border are presented. Scale bar, 50 μm. (C) Analysis of TT1_Ct_ delivery to male and female mice cortex after 5 h of ischemia. Bio-TT1_Ct_ and Bio-TMyc were injected as before and detected in the contralateral region of coronal sections by Fluorescein Avidin D (green). Neuronal marker NeuN (magenta) is also shown. Peptide delivery was observed in neuronal (asterisks) and non-neuronal cells (arrowheads). Representative confocal microscopy images of cortical areas correspond to single sections. Scale bar, 20 μm.

Our next objective was to investigate if TT1_Ct_ could counteract brain degeneration and interfere TrkB-T1/GFAP expression when excitotoxicity occurs *in vivo*. First, we demonstrated that biotinylated TT1_Ct_ (Bio-TT1_Ct_) and Bio-TMyc (3 nmol/g), intravenously (i.v.) injected into undamaged animals, were able to cross the BBB and efficiently reach most cortical cells in 30 min, being distributed in cell bodies (asterisks) and projections (arrowheads) (Figure S5). Then, we selected a mouse model of permanent ischemia induced by microvascular photothrombosis, which reproduces embolic or thrombotic occlusion of small arteries often produced in human stroke 62. Photothrombosis causes early breaking of the BBB 63 and damage of cortical motor and somatosensory regions, infarcts being detected after 3 h 38 and reaching maximum volumes by 24 h (Figure 5A). Using confocal microscopy (Figure 5B) or a Cell Observer (Figure S6A), respectively for a detailed analysis or a more general overview, we studied the expression of the TrkB isoforms together with cell-type specific markers in the area of the emerging infarct of animals treated with the control peptide 1 h after damage initiation. We confirmed a strong decrease of TrkB-FL in neurons of the infarcted area, identified by condensed nuclei indicative of cell injury, by 5 h of injury. In contrast, levels of TrkB-T1 and GFAP already increased in the infarcted area at this early time of damage, particularly in the interface of ischemic and non-ischemic tissue which might correspond to the emerging glial scar. Similar results were obtained in animals injected as before with vehicle (Figure S6B). Next, we analyzed Bio-TMyc and Bio-TT1_Ct_ distribution in male and female mice cortex 5 h post-stroke, detecting them in both neuronal (arrows), and non-neuronal cells (asterisks) of the contralateral hemisphere (Figure 5C). Peptide detection at least 4 h after administration suggests a relative stability in brain.

**Figure 6:**
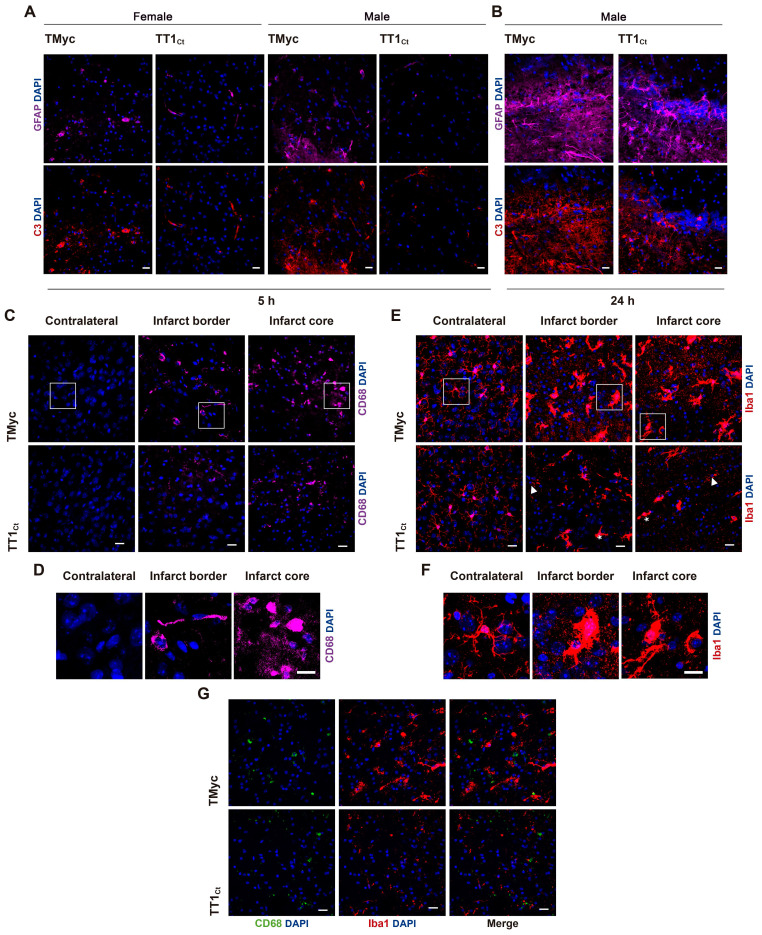
** Treatment with TT1_Ct_ prevents reactive gliosis at early and late times of ischemic damage.** (A) Coronal sections of male and female mice injected with TMyc or TT1_Ct_ (3 nmol/g) after 5 h of insult were stained with antibodies for C3 (red), GFAP (magenta) and DAPI to detect reactive astrocytes. Representative maximum intensity projection confocal images from the infarct border are shown. Scale bar, 20 μm. (B) Coronal sections of male mice treated with TMyc or TT1_Ct_ (3 nmol/g) after 24 h of insult were stained as above. Representative maximum intensity projection of confocal images from the infarct border are shown. Scale bar, 20 μm. (C) Coronal sections of male mice injected with TMyc or TT1_Ct_ (3 nmol/g) after 24 h of insult were stained with an antibody for CD68 (magenta) and DAPI to detect microglia and macrophage inflammatory state. Representative maximum intensity projection of confocal images from the contralateral cortex, infarct core and infarct border are shown. Scale bar, 20 μm. (D) Detail of cell morphology in selected areas indicated in panel C, corresponding to animals injected with TMyc as above indicated. Scale bar, 20 μm. (E) Coronal sections of male mice injected with TMyc or TT1_Ct_ (3 nmol/g) after 24 h of insult were stained with antibody for Iba1 (red) and DAPI to detect microglia and macrophage inflammatory state. Representative maximum intensity projection of confocal images of the contralateral cortex, infarct core and infarct border are shown. Reactive microglia (asterisks) and resting microglia (arrows) were detected. Scale bar, 20 μm. (F) Detail of cell morphology in selected areas indicated in panel E, corresponding to animals injected with TMyc as above indicated. Scale bar, 20 μm. (G) Double immunohistochemistry with CD68 (green) and Iba1 (red) antibodies of the infarct core of male mice injected with TMyc or TT1_Ct_ as above to analyze possible signal overlapping. Representative maximum intensity projection of confocal images are shown. Scale bar, 20 μm.

To investigate TT1_Ct_ effects on the glial subpopulation in stroke, we first evaluated GFAP and complement component 3 (C3) co-staining after 5 h (Figure 6A) or 24 h (Figure 6B) of injury. C3 is a marker of inflammatory reactive astrocytes which, among other features, lose the capacity to support neuronal survival and synaptogenesis, and induce death of neurons and mature differentiated oligodendrocytes 7. TMyc-treated animals had most GFAP+ cells co-stained by the C3 antibody at the infarct boundary, both at 5 and 24 h of damage. Moreover, the number of cells identified as GFAP+/C3+ strongly increased over time. In contrast, early after injury, TT1_Ct_ caused a strong decrease in GFAP+/C3+ cells. In addition to an early effect on glial scar formation, we investigated a possible TT1_Ct_ impact on macrophage infiltration and microglia activation in injured brain (Figure 6C-G). Protein CD68 (ED1), expressed in macrophages, activated microglia and other cell types, was detected in the infarct border and core of animals treated with TMyc, while it was absent of the contralateral region (Figure 6C). Most CD68+ cells had a rounded morphology compatible with macrophages, although more elongated cells were also detected (Figure 6D). TT1_Ct_ treatment greatly decreased CD68+ cells in the infarct and surrounding tissues, in parallel with reduced immunoglobulin infiltration (Figure S7), produced as early as 2.5 h after injury by BBB damage 45. Expression of Iba1, a protein present in microglia and circulating macrophages (Figure 6E), was detected in cells with ramified morphology characteristic of resting microglia located in the contralateral region of TMyc and TT1_Ct_-treated animals (Figure 6E-F). Iba1 upregulation was observed in ischemic brain of TMyc-treated animals accompanied by morphological cellular changes indicative of hypertrophic activated microglia. TT1_Ct_ limited Iba1 upregulation in reactive microglia and decreased these cells presence in damaged tissue (Figure 6E). Double immunostaining showed very little CD68/Iba1 signal overlapping (Figure 6G). Altogether, these results show the importance of TrkB-T1 in glial scar formation and inflammatory response after ischemia, as well as the possibility to restrain these processes by using brain-accessible peptide TT1_Ct_.

**Figure 7:**
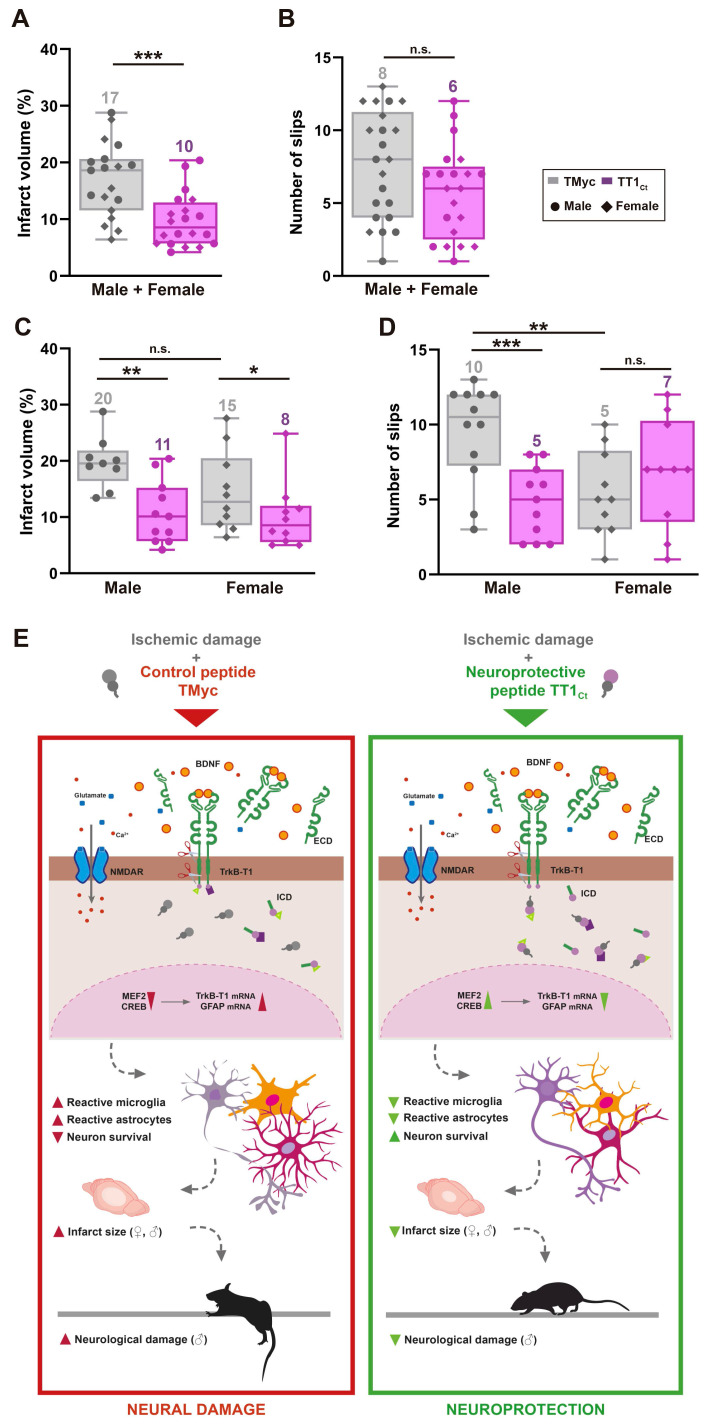
** Treatment with TT1_Ct_ reduces infarct volume and motor coordination deficits in animals exposed to permanent ischemia.** (A and C) Infarct volume of animals injected with TMyc or TT1_Ct_ (3 nmol/g) and sacrificed 24 h after damage induction, expressed as a percentage of the hemisphere volume. Individual data and box and whisker plots show interquartile range, median, minimum and maximum values. The mean value for each experimental group is also provided as a number on top of the corresponding plot. Results are given for the whole population (A) or disaggregated according to gender (C). Differences were analyzed by Student's *t*-test (*n* = 20 for A, *n* = 9-10 for C). (B and D) Evaluation of balance and motor coordination. Number of contralateral hind paw slips were measured in male and female animals. As above, results are presented for the whole population (B) or disaggregated according to gender (D). Differences were analyzed by Student's *t*-test (*n* = 21-22 for B, *n* = 10-12 for D). (E) Model proposed for TT1_Ct_ action. In the presence of the control peptide TMyc, ischemic damage induces TrkB-T1 RIP and binding of particular proteins to the isoform-specific C-ter sequence, present in TrkB-T1-ICD or unprocessed full-length protein. This excitotoxicity-induced binding results, by still undefined mechanisms, in shut-off of CREB and MEF2 promoter activities and transcriptional changes, affecting neurons and glial cells. Among other transcripts, the increase in TrkB-T1 and GFAP mRNA levels could contribute to decreased neuronal survival concurrent with increased microglia and astrocyte reactivity, resulting in bigger infarcts and a worse neurological outcome. In contrast, by interfering TrkB-T1 protein interactions, TT1_Ct_ would help to maintain CREB and MEF2 activities and prevent the transcriptional changes induced by excitotoxicity, contributing to neuronal survival and reduced reactive gliosis. Consequently, the infarct size and the neurological damage would be strongly diminished.

Finally, we characterized if TT1_Ct_ effects on reactive gliosis resulted in brain protection after ischemic injury and improved the neurological outcome of affected animals. First, we analyzed TT1_Ct_ effect, administered in male and female animals 1 h after damage induction, on infarct volume (Figure 7A) and neurological damage (Figure 7B) evaluated at 24 h. This setting mimics a clinical situation where neuroprotective molecules are provided after insult onset. TT1_Ct_ (3 nmol/g, i.v.) significantly reduced infarct size (10 ± 1% relative to total hemisphere volume) compared to TMyc (17 ± 2%, ***P < 0.001, *n* = 19; Figure 7A). Notably, TT1_Ct_ administered 10 min after damage had a comparatively modest effect on infarct volume (23%) and neurological damage (29%) (data not shown). Gender disaggregated data showed, for TMyc-treated animals, a tendency to reduced infarct volume in female mice compared to male (15 ± 2% versus 20 ± 1%; *P* = 0.08, *n* = 9: Figure 7C).

However, TT1_Ct_-treatment decreased infarct volume to a similar extent, respectively 42% and 45% in female and male mice, peptide differences being statistically significant for both genders (*P < 0.05 for female, **P < 0.01 for male; Figure 7C). Interestingly, there was a sex-dependent difference in the neurological damage due to ischemia. In male animals, changes in balance and motor coordination established in the beam walking test correlated with injury (Figure 7D). Thus, TMyc-treated mice presented a significant higher number of slips (10 ± 1) compared to TT1_Ct_-treatment (5 ± 1, ***P < 0.001, *n* = 12), representing a 49% recovery. However, TMyc-treated female mice showed more discreet motor deficits than males (5 ± 1 versus 10 ± 1, **P < 0.01, *n* = 10), hindering the observation of a TT1_Ct_ effect with this test and suggesting a sexual dimorphism in the neurological outcome after ischemia.

In conclusion, these results demonstrate that peptide TT1_Ct_ is able to rapidly reach the brain cortex of animals with an intact BBB or those subjected to ischemia, where it can be detected in cortical neurons and astrocytes for at least 4 h. As summarized in Figure 7E, this CPP is neuroprotective both in male and female mice subjected to ischemia, reducing infarct size in parallel to neuroinflammation. Furthermore, TT1_Ct_ improves neurological deficits resulting from ischemia in the male subpopulation. Altogether, these data support the importance of protein interactions established by TrkB-T1 C-ter in reactive gliosis after brain damage, a process that has an impact on neurotrophic-signaling and neuronal survival.

## Discussion

Glial cells reactivity and subsequent inflammation are processes common to stroke 64 and other neurodegenerative conditions 65,66. These cells contribute to neurological diseases by protective and detrimental effects implying complex and heterogeneous changes in cell morphology, gene expression and function. Consequently, glial-targeted neuroprotective therapies able to modulate neuroinflammation are a priority. Receptor TrkB-T1, expressed both in neurons and glial cells, is relevant to neuronal excitotoxicity 9,35 while, in astrocytes, promotes injury and oedema formation after stroke 33. Specific interactions established by TrkB-T1 with still undefined proteins might be important for reactive gliosis and neurotoxicity and, therefore, their modification might prevent such pathological processes and decrease ischemic damage. We have designed brain-accessible peptides containing the TrkB-T1 isoform-specific sequence and show that they modulate reactive gliosis and are strongly neuroprotective against *in vitro* and *in vivo* excitotoxicity.

An important mechanism of TT1_Ct_ action in excitotoxicity is maintenance of CREB/MEF2 activities, essential in neuroinflammation by brain injury 67-69 and neuronal survival/death 51,70. Compared to neurons, CREB role in astrocytes is not completely defined 71 although different adaptative transcriptional programs are regulated in both cell types 72. In excitotoxicity, maintenance of CREB/MEF2 promoter activities by TT1_Ct_ action changes levels of CREB and/or MEF2-regulated mRNAs encoding proteins important for astrocyte function. Thus, TT1_Ct_ induces a strong increase of CREB/MEF2-regulated *Bdnf* transcription 18-20 while it inhibits TrkB-T1 and GFAP expression, suggesting positive and negative effects of these TFs in astrocytes. An inverse association of CREB or MEF2D activation with GFAP expression has been previously described in models of Alzheimer disease 73 or isoflurane neuroprotection in ischemia/reperfusion 74. These results are relevant because GFAP expression levels, alongside astrocyte reactivity, are considered proportional to injury severity 75.

Similar TT1_Ct_ effects were observed in a preclinical stroke model, where excitotoxicity occurs *in vivo*. Very early after ischemic damage (5 h), proteins TrkB-T1 and GFAP increase in some cells of the infarcted area, particularly in the interface between ischemic and non-ischemic tissue. At later times (24 h), GFAP expression further increases in this area in TMyc-treated animals, mostly in C3+ cells indicative of inflammatory astrocytes. In contrast, TT1_Ct_ strongly decreases GFAP+/C3+ cells at both times. Remarkably, TT1_Ct_ also downregulates macrophage entry and IgG infiltration into the injured tissue, indicative of reduced BBB damage. Finally, microglial Iba1 upregulation in the infarct core and the interface between ischemic and non-ischemic tissue is also decreased by TT1_Ct_ action. Globally, these results demonstrate the importance of signaling mediated by TrkB-T1 C-ter in the modulation of the acute inflammatory response after stroke. Complexes formed by unprocessed TrkB-T1 or RIP TrkB-T1-ICD fragment might regulate the expression of proteins central to ischemia such as the truncated receptor itself, GFAP, C3 or Iba1. This regulation might depend on CREB/MEF2 or additional TFs. One possibility is that, similarly to Notch-ICD 76, TrkB-T1-ICD directly interacts with CREB and alters CREB-dependent expression. Anyway, by interfering TrkB-T1-interactions, TT1_Ct_ might prevent excitotoxicity-induced transcriptional changes, resulting in decreased microglia and astrocyte reactivity after ischemia.

To integrally expose specific TrkB-T1-interacting proteins in different types of neural cells and their relevance in receptor-signaling and function, particularly in the context of an excitotoxic response, we performed proteomic analysis of primary cortical cultures, our *in vitro* model of excitotoxicity. Thus, the identified proteins might correspond to those interacting with TrkB-T1 in neurons, astrocytes or both. In the future, it would be interesting to perform similar studies in primary cortical cultures enriched in astrocytes or neurons. Except for RhoGDI, discovered in astrocyte extracts by affinity chromatography with the isoform-specific peptide 29, no other TrkB-T1-interacting proteins had been previously identified. Results presented here demonstrate that Bio-sTT1_Ct_ establishes a specific protein-interacting profile inside neural cells which is susceptible to different conditions. A differential analysis of Bio-sTT1_Ct_-interactions shows that, independently of treatment, this peptide interacts with proteins highly involved in gene expression, including RNA modification or splicing, such as the RNA editing enzyme adenosine deaminase acting on RNA (ADAR1). This protein, highly induced in astrocytes after stroke, exacerbates ischemic-injury via astrocyte-mediated neuronal apoptosis 77. Regarding BDNF treatment, an enrichment of pathways mainly related with vesicle transport and mTOR regulation is observed. Thus, BDNF induces a significant increase in Bio-sTT1_Ct_-binding to neuron-enriched cholesterol 24-hydroxylase (CYP46A1), which controls cholesterol turnover and synthesis of a product that contributes to ischemic damage 78. Finally, excitotoxicity also produces an enrichment in pathways related with vesicle transport and, more specifically, interrelated pathways involved in axon guidance, innate immune response, including L1CAM interactions, or RhoGTPases-signaling. This protein family, together with RhoGTPase-related proteins, connects activation of surface receptors to cytoskeleton organization, coordinating processes such as cell migration and polarity, cell cycle progression, transcription or neuronal survival/death 30. RhoGTPases are also central to development of the ischemic damage, particularly in morphological changes associated with reactive gliosis 79.

One protein involved in RhoGTPase-signaling that differentially and significantly interacts with Bio-sTT1_Ct_ in excitotoxicity is p21-activated kinase 5 (PAK5), which belongs to a family of downstream effectors of Rac1/Cdc42 RhoGTPases. PAK proteins participate in cytoskeleton remodeling, promotion of inflammatory cells transcription, and survival 80. Interestingly, microglia use a P2Y12R-Rac-PAK-F-actin pathway to alleviate neurons from cytotoxic protein aggregates and regulate delivery of healthy mitochondria to burdened neurons through tunnelling nanotubes (TNTs) 81. Another protein central to TNT formation is actin-related protein 2/3 (Arp2/3), a multiprotein complex that modulates actin polymerization/depolymerization downstream of Rac. Notably, two Arp2/3 subunits (Actr2/Arp2 and Arpc3/p21-Arc) differentially interact with Bio-sTT1_Ct_ in excitotoxicity. Finally, activation in axons of PAK5 synthesis and signaling after spinal cord injury or cerebral ischemia protects neurons from an axonal energy crisis by reprogramming mitochondrial trafficking and anchoring 82. Other RhoGTPases-related proteins that differently interact with Bio-sTT1_Ct_ in excitotoxicity are the putative receptor of complement component 1q (C1qbp), C1q being secreted by reactive microglia and involved in induction of neurotoxic reactive astrocytes 7, proteins Nup98 and Ranbp2, important for maintenance and/or assembly of the nuclear pore complex and nucleocytoplasmic transport 83, and engulfment and cell motility 2 (Elmo2) protein, an upstream Rac1 regulator required for phagocytosis and cell migration 84. Altogether, our results strongly suggest that excitotoxicity might alter specific interactions established by TrkB-T1 with proteins involved in RhoGTPase-signaling, critical to cell morphology, neuronal survival and reactive gliosis. While some TrkB-T1-interactions are inhibited in excitotoxicity, others are strongly promoted. Displacement by Bio-sTT1_Ct_ of the latter could interfere pathways associated to neuroinflammation and neurotoxicity, thus leading to neuroprotection.

The complexity of TrkB-T1 interactome allows us to propose that a combination of mechanisms affecting neurons and astrocytes alike could be responsible for TT1_Ct_ neuroprotective actions, demonstrated in excitotoxicity *in vitro* and *in vivo*. In cortical cultures, the peptide is strongly neuroprotective when used at moderate concentrations (15 µM) and added before or at the time of the excitotoxic stimulus. This result is important from a preclinical and clinical perspective because, differently from cultured cells, excitotoxicity is not synchronous *in vivo*. By the time treatment is initiated in the ischemia model, penumbra neurons, an important therapeutic target, would be exposed, if not properly protected, to secondary damage associated to infarct expansion. In fact, TT1_Ct_-treatment 1 h after ischemic injury leads to a significant reduction of the infarct volume both in male and female mice, a result particularly relevant considering that our preclinical model has a relatively narrow ischemic penumbra 85. It will be interesting to analyze in the future if treatment with TT1_Ct_ can be delayed further after damage. Interestingly, results obtained in male and female mice reveal a sexual dimorphism relative to the effect of the ischemic damage on motor coordination and balance. As previously described 38,45, males show pronounced deficits which can be improved by TT1_Ct_, while females are less compromised despite having similar infarct volumes. Sexual dimorphism after CNS injury has been previously observed in other studies 86,87, and has been related to a neuroprotective estrogen effect or a decreased neuroinflammatory response in female mice 88. Therefore, future studies on the pathophysiology of ischemic brain damage and testing of therapeutic compounds need to consider the impact of biological sex.

Clinical translation of above results could be limited by a predictable low plasma stability of TT1_Ct_, conditioning peptide brain bioavailability and efficacy. However, as previously described in patients treated with nerinetide, a low CPP half-life is not necessarily related to a lack of neuroprotection. Nerinetide is a Tat-derived CPP (20 aa) that dissociates the ternary complex formed by NMDAR-GluN2B subunits with scaffold protein PSD-95 and neuronal nitric oxide synthase (nNOS) 89. In an excitotoxic context, this peptide reduces nNOS activation, nitric oxide (NO) production and oxidative damage, resulting in neuroprotection in different preclinical stroke models 89-91. Remarkably, in the course of a phase 3 clinical trial for acute ischemic stroke, nerinetide-treatment had a clinical benefit in patients undergoing endovascular therapy without concurrent or previous thrombolytic treatment, even when the plasma half-life of nerinetide was below 20 min 42. One possibility is that CPP stability once it reaches the brain tissue is higher compared to plasma, as suggested by our results showing the presence of Bio-TMyc and Bio-TT1_Ct_ in male and female brain cortex at least 4 h after administration. Another relevant issue concerning clinical translation is the appropriate time of CPP treatment after onset of stroke symptoms. Recently published results from the FRONTIER clinical assay (NCT0231544) have shown that administration of nerinetide by paramedics before hospital arrival might benefit patients with acute ischemic stroke as an adjunct to reperfusion therapies if applied within 3 h of symptoms onset 43. In fact, in this trial, nerinetide treatment of suspected stroke began a median of 64 min (IQR 47-100) from symptoms onset. Furthermore, a *post-hoc* metanalysis of data collected from three completed randomized nerinetide trials enrolling patients up to 12 h after stroke onset further supports the importance of early peptide treatment followed by reperfusion to obtain a clinically significant benefit over several outcome measures 92.

The peptide TT1_Ct_ described in this work has multimodal effects in the damaged brain, affecting neurons as well as glial cells, and might be relevant for the design of novel therapies for human stroke and many other neurological conditions associated with excitotoxicity and neuroinflammation.

## Supplementary Material

Supplementary methods, figures and tables.

## Abbreviations

Arp2/3: actin-related protein 2/3
BBB: blood-brain barrier (BBB)
BDNF: brain-derived neurotrophic factor
C1q: complement component 1q
C3: complement component 3
CPP: cell-penetrating peptide
CREB: cAMP response element-binding protein
CYP46A1: cholesterol 24-hydroxylase
DIVs: days *in vitro*
Elmo2: engulfment and cell motility 2 protein
GABA: gamma aminobutyric acid
GAPDH: glyceraldehyde-3-phosphate dehydrogenase
GFAP: glial fibrillary acidic protein
GO: gene ontology
ICDs: intracellular domains
MEF2: myocyte enhancer factor 2
MPs: metalloproteinases
NDDs: neurodegenerative diseases
NMDARs: N-methyl-D-aspartate receptors
NPC: nuclear pore complex
n.s.: non-significant
NSE: neuronal specific enolase
PAK5: p21-activated kinase 5
PCA: Principal Component Analysis
RhoGDI1: RhoGDP dissociation inhibitor 1
RIP: regulated intermembrane proteolysis
TFs: transcription factors
TK: tyrosine kinase
TNTs: tunnelling nanotubes
TrkB: tropomyosin-related kinase B receptor
TrkB-FL: full-length TrkB
