# Supplementary material for: A brain-accessible peptide modulates stroke inflammatory response and neurotoxicity by targeting BDNF-receptor TrkB-T1 specific interactome
Source: Theranostics. 2025 Mar 21;15(10):4654–72. doi: 10.7150/thno.111272 (PMC11984388; doi:10.7150/thno.111272)
Supplement: Supplementary file 1 — Supplementary methods, figures and tables. [file thnov15p4654s1.pdf]

Supplementary Material

Supplementary Figures

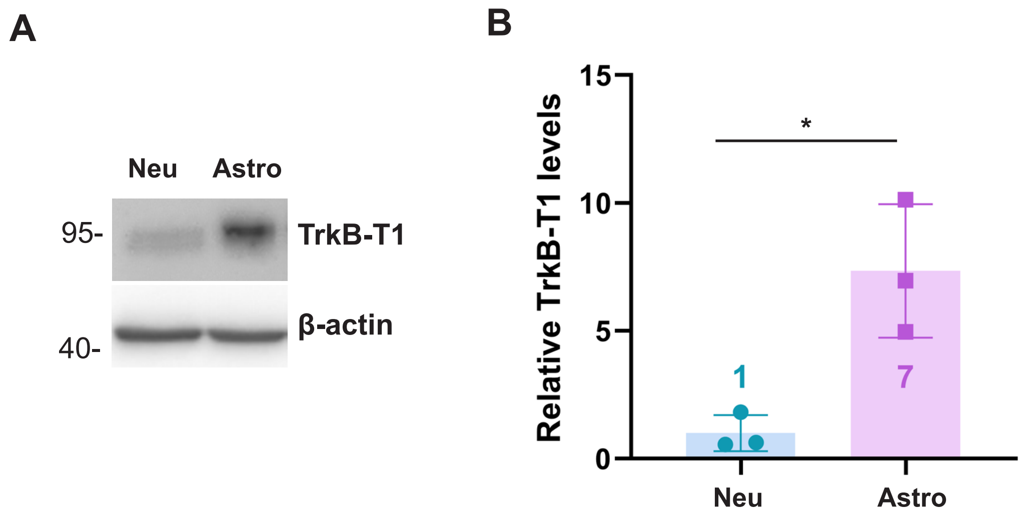

Figure S1. Comparison of TrkB-T1 protein levels in primary cultures of mixed cortical cells or astrocytes. (A) Representative immunoblot analysis of equivalent amounts of protein lysates corresponding to cultures enriched, respectively, in neurons (Neu) or astrocytes (Astro). Levels of the TrkB-T1 isoform and ß-actin, used for normalization, are shown. (B) Quantitation by densitometric analysis of normalized TrkB-T1 levels showing the fold-increase in astrocytes compared to the mixed cultures. Means ± SEM are presented and differences were analyzed by Student's t-test (n = 3)

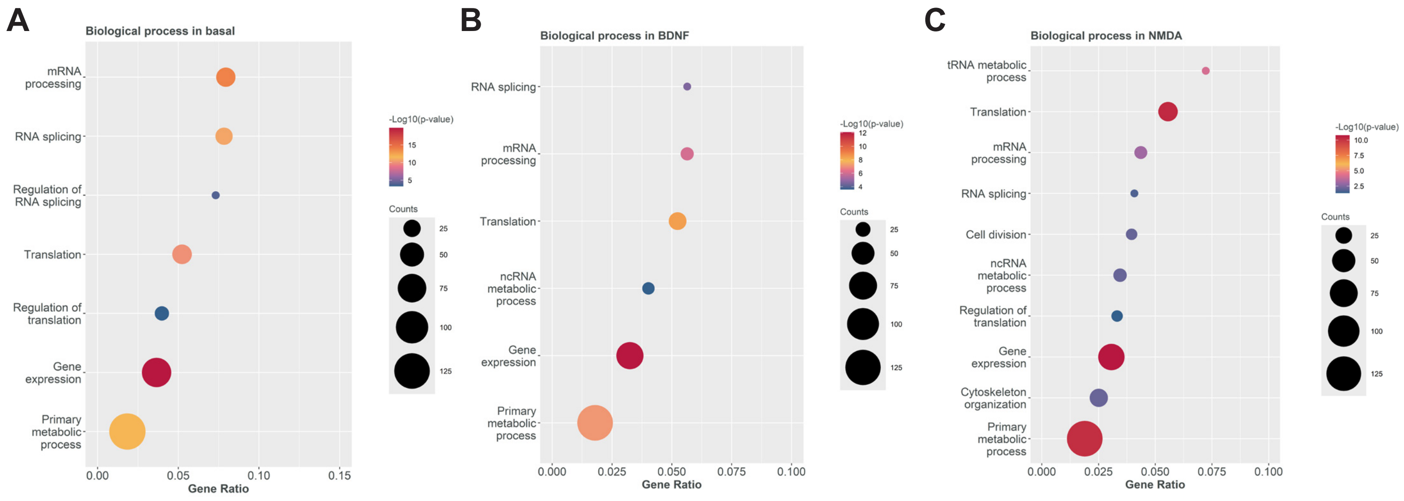

Figure S2. Biological processes associated with Bio-sTT1$_{Ct}$ protein interactions. Representation of the most relevant Gene Ontology (GO) terms related to biological processes for selected proteins interacting with Bio-sTT1$_{Ct}$ in basal conditions (A) or after treatment with BDNF (B) or NMDA (C). The dot size represents the number of proteins from our data set related to each process. Dots are colored according to their statistical significance, which is set by a color scale referring to -log10 (adjusted p-value).

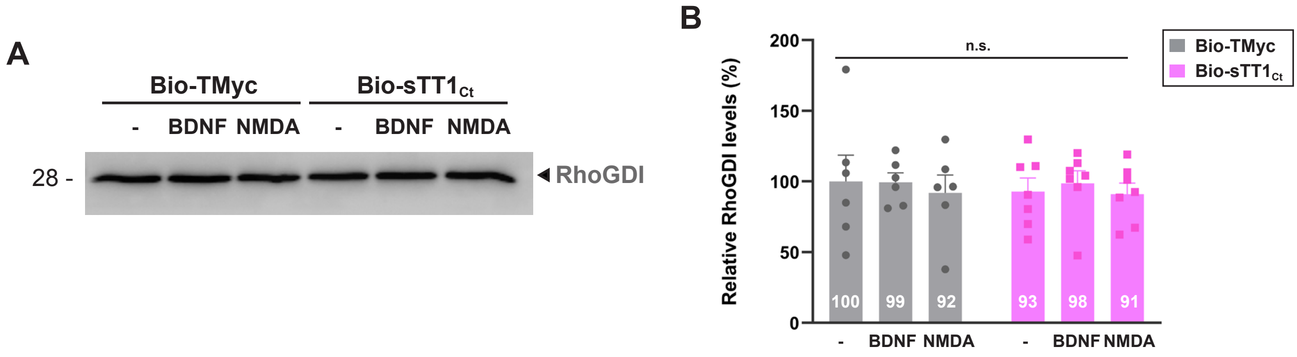

Figure S3. Total RhoGDI1 levels are not affected by BDNF or NMDA treatment. (A) Western blot analysis of cortical cultures incubated with Bio-sTT1$_{Ct}$ or Bio-TMyc (25 µM) for 30 min before treatment with BDNF (100 ng/ml) or NMDA for 30 min. A representative experiment is presented showing RhoGDI levels in total lysates. (B) Quantitation by densitometric analysis of RhoGDI total levels. Means ± SEM are presented relative to basal conditions with Bio-TMyc (100%). Data were analyzed by ANOVA test followed by *post-hoc* Tukey's test, n = 6-7.

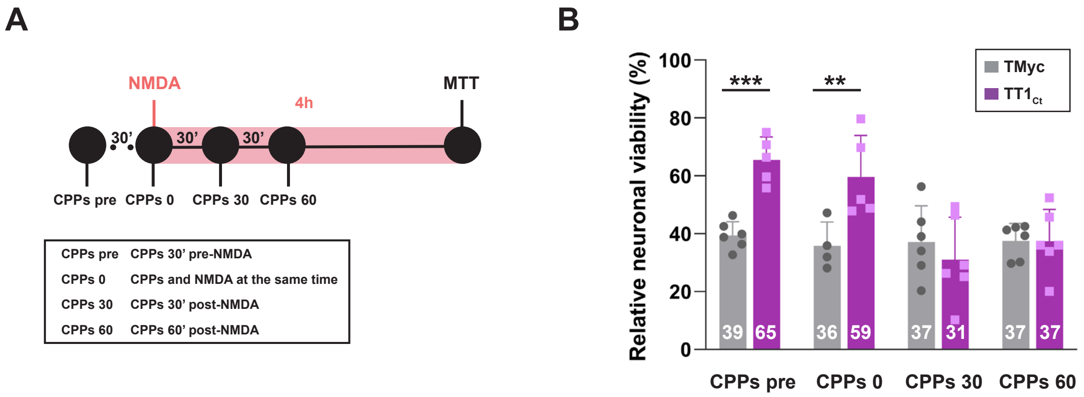

Figure S4. Neuroprotection due to $TT1_{Ct}$ preincubation is maintained when applied at the time of damage induction. (A) MTT assay design to evaluate $TT1_{Ct}$ neuroprotection in primary cortical cultures treated with CPPs at different time points of damage induction. TMyc or $TT1_{Ct}$ were added 30' before (CPPs pre), at the same time (CPPs 0), 30 min (CPPs 30) or 60 min (CPPs60) after treatment with NMDA (100 µM). Neuronal viability was established after 4 h of excitotoxicity. (B) Neuronal viability in cultures treated as above indicated. Individual results and means ± SEM are presented relative to the values obtained for the untreated cells (100%). Data were analyzed using two-way ANOVA test followed by *post hoc* Bonferroni test, n = 4-8.

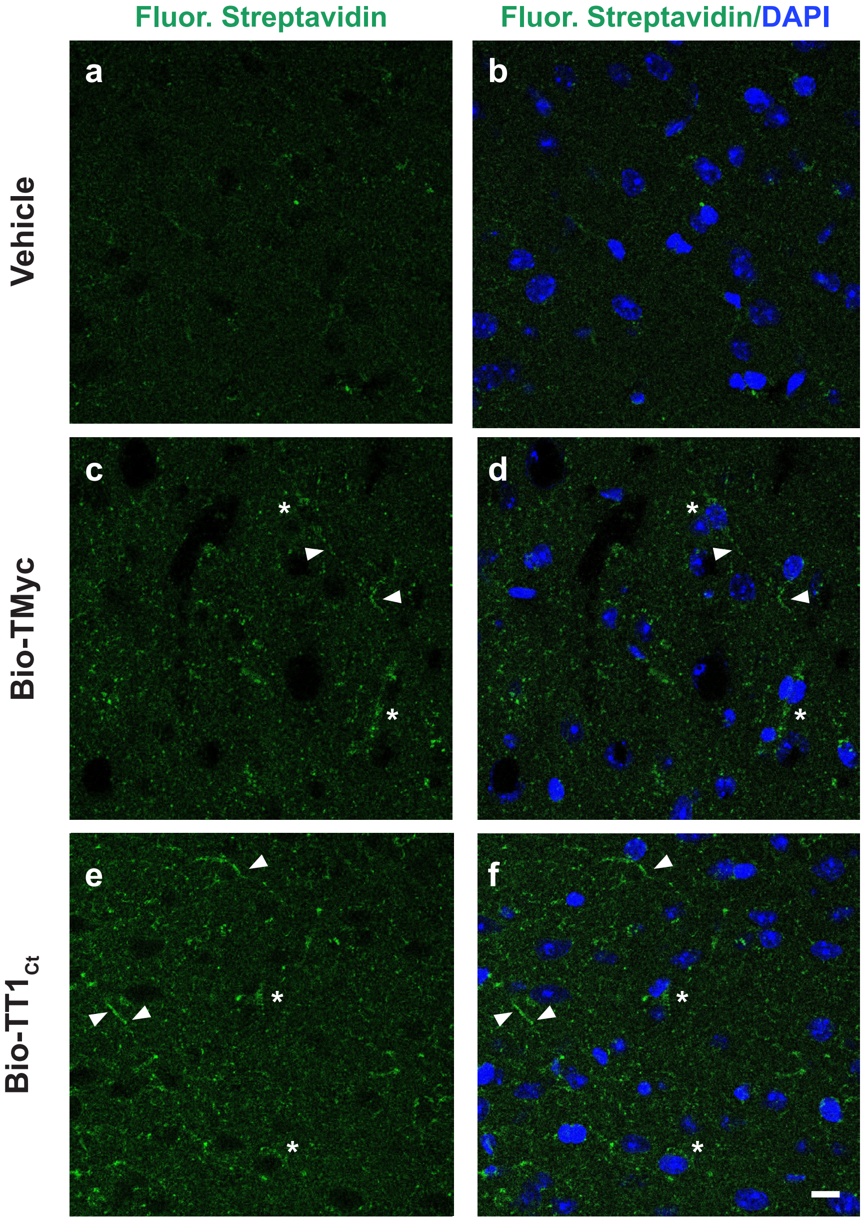

Figure S5. Bio-TT1$_{Ct}$ and Bio-TMyc can cross the BBB of undamaged animals. Analysis of biotinylated-peptide delivery to undamaged male mice cortex. Bio-TT1$_{Ct}$ and Bio-TMyc (3 nmol/g) were injected, and animals were sacrificed 30 min after. Detection was made in coronal sections by Fluorescein Avidin D (green). Peptide delivery was observed in cell projections (arrowheads) and bodies (asterisks) of cortical cells. Representative confocal microscopy images of cortical areas correspond to single sections. Scale bar, 20 µm.

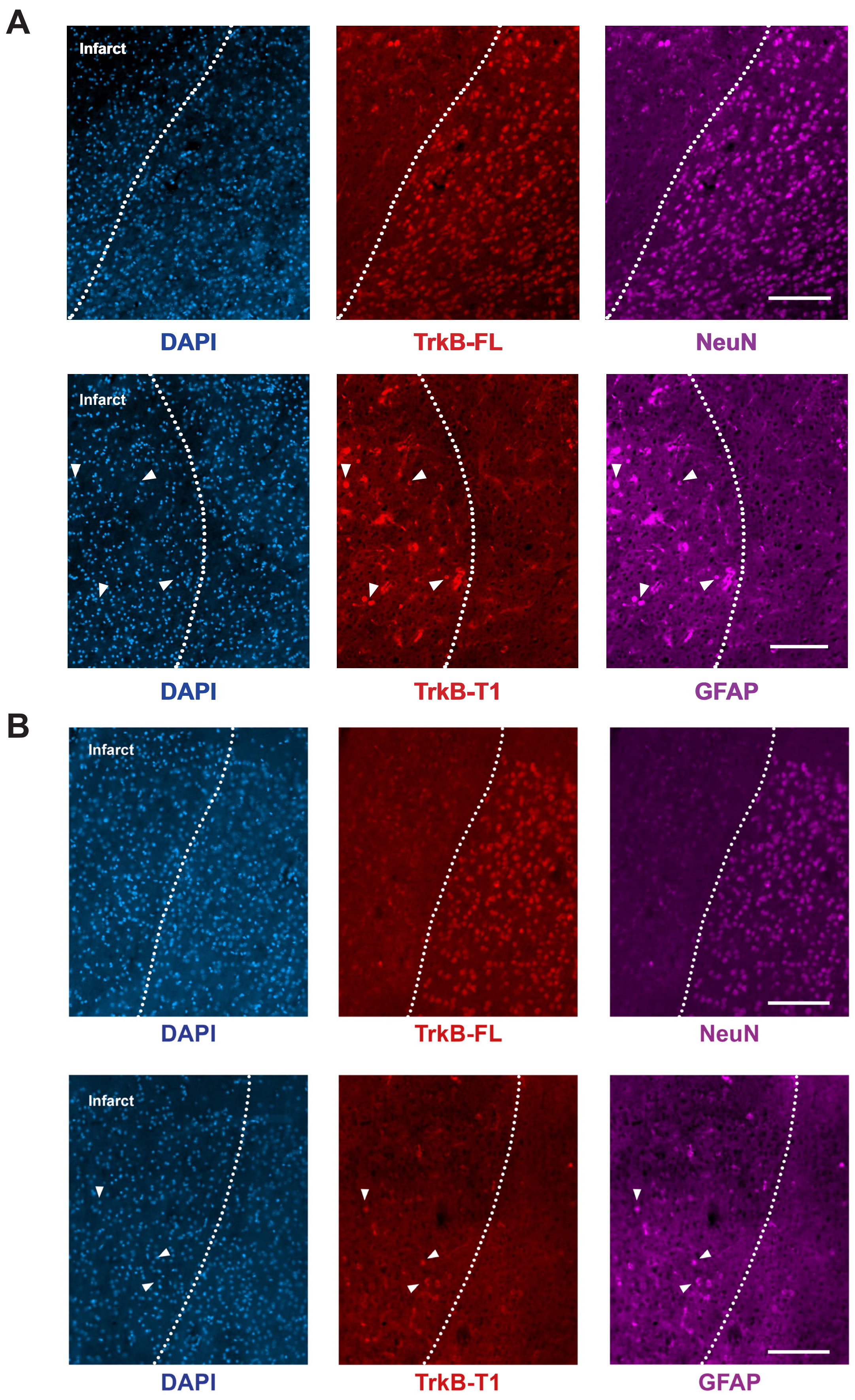

Figure S6. Analysis of the expression of TrkB isoforms and cell-specific markers in damaged brain. Immunohistochemistry of brain coronal sections prepared from male animals i.v. injected with Bio-TMyc (3 nmol/g) (A) or vehicle (B) one hour after damage initiation and sacrificed after 5 h of injury. Sections were stained with isoform-specific TrkB antibodies (TrkB-FL and TrkB-T1), NeuN, GFAP and DAPI. Representative images of cortical areas of the infarct border, obtained with a Cell Observer, are shown. Arrows indicate concurrent increased expression of TrkB-T1 and GFAP in astrocytes. Scale bar, 50 µm.

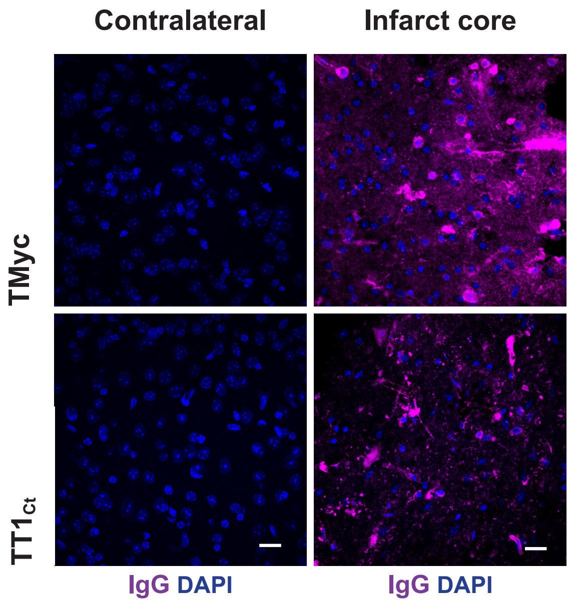

Figure S7. Leakage of mouse immunoglobulins to the brain cortex due to BBB breakage is decreased in TT1$_{Ct}$-treated animals. Brain coronal sections of male animals sacrificed 24 h after insult were analyzed by immunohistochemistry with an anti-mouse secondary antibody. Heavy staining of blood vessels, infiltrated cells and high backgrounds were observed in the ischemic brain of animals injected with the control peptide and decreased in TT1$_{Ct}$-treated animals. Representative images correspond to single sections. Scale bar, 20 µm.

Table S1. Key reagents and resources with indication of sources and identifiers.

REAGENT or RESOURCE	SOURCE	IDENTIFIER
Antibodies		
Mouse monoclonal anti-β-**Actin**	Sigma-Aldrich	Cat#A5441<br>RRID: AB_10920058
Rabbit polyclonal anti-**C3d**	Dako	Cat#A0063<br>RRID: AB_578478
Mouse monoclonal anti-**CD68 (ED1)**	Millipore	Cat#MAB1435;<br>RRID: AB_177576
Rabbit polyclonal anti-**pS133-CREB**	Millipore	Cat#06-519;<br>RRID:AB_310153
Rabbit monoclonal ani-**GFAP**	Millipore	Cat#MAB360<br>RRID:AB_11212597
Mouse monoclonal anti-**GFAP**	Millipore	Cat#G6171<br>RRID:AB_1840893
Rabbit polyclonal anti-**Iba1**	Wako	Cat#019-19741<br>RRID: AB_839504
Mouse monoclonal anti-**MEF2D**	BD Biosciences	Cat# 610774,<br>RRID:AB_398095
Mouse monoclonal anti-RBFOX3/**NeuN**	Novus	Cat#NBP1-92693SS;<br>RRID:AB_1103747
Rabbit polyclonal anti-neuronal-specific enolase (**NSE**)	Millipore	Cat#AB951;<br>RRID:AB_92390
Rabbit polyclonal anti N-ter region of **RhoGDI**	Santa Cruz	Cat#sc-360<br>RRID: AB_2227516
Rabbit polyclonal anti-**TrkB-FL (C-ter region)**	Santa Cruz	Cat#sc-11;<br>RRID:AB_632554
Rabbit polyclonal anti-**TrkB-T1** (isoform-specific C-ter)	Custom made	
Goat anti-mouse IgG Alexa Fluor 546	Molecular Probes	Cat#A-11030;<br>RRID:AB_144695
Goat anti-mouse IgG Alexa Fluor 647	Molecular Probes	Cat#A-21236<br>RRID:AB_2535805
Goat anti-rabbit IgG Alexa Fluor 488	Molecular Probes	Cat# A11034<br>RRID: AB_2576217
Goat anti-rabbit IgG Alexa Fluor 546	Molecular Probes	Cat# A11035<br>RRID: AB_143051
Donkey anti-rabbit IgG-heavy and light chain-HRP	Bethyl	Cat#A120-108P;<br>RRID:AB_10892625
Donkey anti-mouse IgG-heavy and light chain-HRP	Bethyl	Cat#A90-137P;<br>RRID:AB_1211460
Chemical and biological products		
**Ara C** (used at 10 µM)	Sigma-Aldrich	Cat#C1768;
**B27** serum free supplement	Thermo Fisher	Cat#17504044
Recombinant human/murine/rat brain-derived neurotrophic factor (**BDNF**) (used at 100 ng/ml)	PeproTech	Cat#450-02
**DAPI** (used at 0.5 or 5 µg/ml)	Molecular Probes	Cat#D1306
Human epidermal growth factor recombinant prot. (**EGF**)	Thermo Fisher	Cat# PHG0311
Human basic fibroblast growth factor recomb. prot. (b**FGF**)	Thermo Fisher	Cat#13256-029
**Fluorescein Avidin** D	Vector Laboratories	Cat#A 2001
**Glycine** (used at 10 µM)	Bio-Rad	Cat#161-0718
**Laminin** (used at 4 µg/ml)	Sigma-Aldrich	Cat#L2020
**MTT** (used at 0.5 mg/ml)	Sigma-Aldrich	Cat#M5655
**NMDA** (used at 100 µM)	Tocris	Cat#0114

**PhosSTOP** phosphatases inhibitor cocktail tablets	Roche	Cat#04 906 837 001
**Poly-L-Lysine** (used at 100 µg/ml)	Sigma-Aldrich	Cat#P1524
**Prolong** Diamond antifade reagent	Molecular Probes	Cat#P36970
Complete **protease inhibitor** cocktail tablets	Roche	Cat#11 697 498 001
**Rose Bengal** (used at 20 mg/kg)	Sigma-Aldrich	Cat#R3877
**Streptavidin** resin	GenScript	Cat#L00353
**TTC** (used at 2%)	Sigma-Aldrich	Cat#T8877
Peptides		
**Bio-sTT1$_{Ct}$** (Biotin-*YGRKKRRQRRR*FVLFHKIPLDG)	GenScript	N/A
**Bio-TMyc** (Biotin-*YGRKKRRQRRR*AEEQKLISEEDLLR)	GenScript	N/A
**Bio-TT1$_{Ct}$** (Biotin-*YGRKKRRQRRR*PPFVLFHKIPLDG)	GenScript	N/A
**TMyc** (*YGRKKRRQRRR*AEEQKLISEEDLLR)	GenScript	N/A
**TT1$_{Ct}$** (*YGRKKRRQRRR*PPFVLFHKIPLDG)	GenScript	N/A
Primer sequences for real-time PCR		
GluN1-F (all isoforms): TCCACCAAGAGCCCTTCGTG	Sigma-Aldrich	N/A
GluN1-R (all isoforms): GCCCCGTACAGATCACCTTC	Sigma-Aldrich	N/A
GluN2A-F: ACGACTGGGACTACAGCCTG	Sigma-Aldrich	N/A
GluN2A-R: CTTCTCTGCCTGCCCATAGC	Sigma-Aldrich	N/A
TrkB-FL-F (isof. spec.): TATCTTCACCCACCTCAAAC	Sigma-Aldrich	N/A
TrkB-FL-R (isof. spec.): GAGAGACTTGACCTGAGCAC	Sigma-Aldrich	N/A
TrkB-T1-F (isof. spec.): GGGGCTGTGCTGCTTGGT	Sigma-Aldrich	N/A
TrkB-T1-R (isof. spec.): GCTGCGGACATCTTTGGAGA	Sigma-Aldrich	N/A
BDNF-F (all isoforms): TGGCTGACACTTTTGAGCAC	Sigma-Aldrich	N/A
BDNF-R (all isoforms): GTTTGCGGCATCCAGGTAAT	Sigma-Aldrich	N/A
GAPDH-F: TTGCCATCAACGACCCCTTC	Sigma-Aldrich	N/A
GAPDH-R: GCCTTGACTGTGCCGTTGAA	Sigma-Aldrich	N/A
NSE-F: ACAGAATGGGGCTGTGTACC	Sigma-Aldrich	N/A
NSE-R: TGGCAACTGTGGGACATGGC	Sigma-Aldrich	N/A
Critical Commercial Assays		
BCA Protein Assay Kit	Thermo Fisher	Cat# 23225
Clarity Western ECL Blotting Substrate	BioRad	Cat# 1705060
Lipofectamine 2000	Life Technologies	Cat#11668019
Experimental Models: Organisms/Strains		
Balb/c inbred mice (Balb/cOlaHsd)	Envigo Rms, Spain S.L.	N/A
Wistar Rat embryos (E18)	In site facility	N/A
Recombinant DNA		
**pCRE** (25-mer oligonucleotide with the sequence of two TrkB CREs subcloned into pTK-Luc)	(Deogracias et al.,2004)	N/A
**pMEF2** (pRSRF; -307 to -242 of *Nur77* promoter with two MEF2 sites subcloned into pGL2-basic)	(Woronicz et al., 1995)	N/A
**pMEF2mut** (two inactivating point mutations in each MEF2 site of pMEF2)	(Woronicz et al., 1995)	N/A
Software		
Adobe InDesign		RRID:SCR_021799
Adobe Photoshop		RRID:SCR_014199
G*Power 3.1.9.7	http://www.gpower.hhu.de/	RRID:SCR_013726
GraphPad Prism		RRID:SCR_002798
ImageJ	https://imagej.net/	RRID:SCR_003070
R Project for Statistical Computing	http://www.r-project.org	RRID:SCR_001905
String	http://string.embl.de/	RRID:SCR_005223
Other		

Tissue-Tek O.C.T Compound	Sakura	Cat#4583
Protran Western blotting nitrocellulose membrane	GE Healthcare	Cat#GE10600002

Table S2. Selected Bio-sTT1$_{Ct}$- interacting proteins showing significantly altered levels after different treatment conditions

BDNF vs basal conditions

Uniprot ID	Gene name	logFC	p-value	Description	Confidence	nº of unique peptides
A0A8I5Y1J2		2.526	0.012	Peptidyl-prolyl cis-trans isomerase OS=Rattus norvegicus OX=10116 GN=ENSRNOG00000067128 PE=3 SV=1	High	2
A0A8I5Y9S3	*Mrpl48*	-1.393	0.025	Mitochondrial ribosomal protein L48 OS=Rattus norvegicus OX=10116 GN=Mrpl48 PE=1 SV=1	High	3
A0A8I5ZLV1	*Mtx2*	2.616	0.031	Metaxin 2 OS=Rattus norvegicus OX=10116 GN=Mtx2 PE=1 SV=1	High	2
A0A8I5ZS37	*Gak*	1.724	0.028	Cyclin G associated kinase OS=Rattus norvegicus OX=10116 GN=Gak PE=1 SV=1	High	3
A0A8I5ZTG0	*Cdc23*	-0.776	0.040	CDC23 (Cell division cycle 23, yeast, homolog), isoform CRA_b OS=Rattus norvegicus OX=10116 GN=Cdc23 PE=1 SV=1	High	6
A0A8I6A326	*Rbm6*	-1.285	0.011	RNA binding motif protein 6 OS=Rattus norvegicus OX=10116 GN=Rbm6 PE=1 SV=1	High	3
A0A8I6A9H6	*Rnps1*	1.442	0.044	RNA-binding protein with serine-rich domain 1 OS=Rattus norvegicus OX=10116 GN=Rnps1 PE=3 SV=1	High	3
A0A8I6AKZ9	*Actr1b*	1.323	0.006	Actin related protein 1B OS=Rattus norvegicus OX=10116 GN=Actr1b PE=1 SV=1	High	2
A0A8I6AN44	*Nfasc*	-1.092	0.027	Neurofascin OS=Rattus norvegicus OX=10116 GN=Nfasc PE=3 SV=1	High	9
A0A8I6GIS7	*Relch*	-1.207	0.031	RAB11 binding and LisH domain, coiled-coil and HEAT repeat containing OS=Rattus norvegicus OX=10116 GN=Relch PE=1 SV=1	High	2
A0A8L2RBV8	*Rpl39-ps13*	0.864	0.038	Ribosomal protein L39 OS=Rattus norvegicus OX=10116 GN=Rpl39 PE=1 SV=1	High	2
F7EN52	*Cyp46a1*	2.113	0.027	Cytochrome P450, family 46, subfamily a, polypeptide 1 OS=Rattus norvegicus OX=10116 GN=Cyp46a1 PE=3 SV=3	High	3
P62893	*Rpl39*	0.864	0.038	60S ribosomal protein L39 OS=Rattus norvegicus OX=10116 GN=Rpl39 PE=1 SV=2	High	2
Q5RJT2	*Ftsj3*	1.666	0.040	pre-rRNA 2'-O-ribose RNA methyltransferase FTSJ3 OS=Rattus norvegicus OX=10116 GN=Ftsj3 PE=1 SV=1	High	3
Q66H79	*Trim32*	1.694	0.044	Tripartite motif protein 32 OS=Rattus norvegicus OX=10116 GN=Trim32 PE=1 SV=1	High	2

NMDA vs BDNF conditions

Uniprot ID	Gene name	logFC	p-value	Description	Confidence	nº of unique peptides
A0A0G2JYD8	*Capn5*	1.827	0.008	Calpain 5 OS=Rattus norvegicus OX=10116 GN=Capn5 PE=1 SV=2	High	3
A0A8I5ZQN4	*Myo1c*	0.906	0.035	Myosin 1C OS=Rattus norvegicus OX=10116 GN=Myo1c PE=1 SV=1	High	7
A0A8I6G6A0	*Gna11*	1.596	0.039	G protein subunit alpha 11 OS=Rattus norvegicus OX=10116 GN=Gna11 PE=1 SV=1	High	4
A0A8L2R9X1	*Abhd6*	2.091	0.024	Abhydrolase domain containing 6, acylglycerol lipase OS=Rattus norvegicus OX=10116 GN=Abhd6 PE=1 SV=1	High	2
A2VCW8	*Septin7*	0.863	0.042	Septin OS=Rattus norvegicus OX=10116 GN=Septin7 PE=1 SV=1	High	10
B2GUZ3	*Mthfd1l*	1.816	0.045	formate--tetrahydrofolate ligase OS=Rattus norvegicus OX=10116 GN=Mthfd1l PE=1 SV=1	High	5
D4A280	*Pak5*	2.383	0.046	Serine/threonine-protein kinase PAK 5 OS=Rattus norvegicus OX=10116 GN=Pak5 PE=1 SV=1	High	2

NMDA vs basal conditions

Uniprot ID	Gene name	logFC	p-value	Description	Confidence	nº of unique peptides
A0A1W2Q6D9	*Otud6b*	1.158	0.027	ubiquitinyl hydrolase 1 OS=Rattus norvegicus OX=10116 GN=Otud6b PE=1 SV=2	High	2
A0A8I5ZN55	*Ago2*	-1.303	0.048	Protein argonaute-2 OS=Rattus norvegicus OX=10116 GN=Ago2 PE=1 SV=1	High	6
A0A8I5ZNV8	*Pcnp*	-1.018	0.034	PEST proteolytic signal-containing nuclear protein OS=Rattus norvegicus OX=10116 GN=Pcnp PE=1 SV=1	High	4
A0A8I6A326	*Rbm6*	-1.589	0.034	RNA binding motif protein 6 OS=Rattus norvegicus OX=10116 GN=Rbm6 PE=1 SV=1	High	3
A0A8J8YFZ4	*Ptbp1*	-2.049	0.013	Polypyrimidine tract-binding protein 1 OS=Rattus norvegicus OX=10116 GN=Ptbp1 PE=1 SV=1	High	6
A0A8L2R9X1	*Abhd6*	1.798	0.033	Abhydrolase domain containing 6, acylglycerol lipase OS=Rattus norvegicus OX=10116 GN=Abhd6 PE=1 SV=1	High	2
D4A280	*Pak5*	2.460	0.038	Serine/threonine-protein kinase PAK 5 OS=Rattus norvegicus OX=10116 GN=Pak5 PE=1 SV=1	High	2

Uniprot ID and gene name are presented for each protein, together with its logFC obtained after comparison of the two indicated conditions. Individual *P*-values for each protein are also shown. Protein description together with quality parameters of detection (confidence and nº of unique peptides) are also shown.

Table S3. ***Arhgdia*** **(RhoGDI1) and the group of selected Bio-TT1$_{Ct}$-interacting proteins included in the Reactome function "Signalling by RhoGTPases"**

Uniprot ID	Gene name	Description	Confidence	nº of unique peptides
A0A0G2K860	*Arhgef12*	Rho guanine nucleotide exchange factor 12 OS=Rattus norvegicus OX=10116 GN=Arhgef12 PE=1 SV=2	High	3
A0A8I5Y1I7	*Scfd1*	Sec1 family domain containing 1 OS=Rattus norvegicus OX=10116 GN=Scfd1 PE=1 SV=1	High	3
A0A8I5YC36	*Ranbp2*	RAN binding protein 2 OS=Rattus norvegicus OX=10116 GN=Ranbp2 PE=1 SV=1	High	4
A0A8I6AHE2	*Dync1i1*	Dynein cytoplasmic 1 intermediate chain 1 OS=Rattus norvegicus OX=10116 GN=Dync1i1 PE=3 SV=1	High	5
A0A8I6ALK1	*Nup98*	Nuclear pore complex protein Nup98-Nup96 OS=Rattus norvegicus OX=10116 GN=Nup98 PE=1 SV=1	High	2
A0A8I6ALV8	*Tuba1a*	Tubulin alpha chain OS=Rattus norvegicus OX=10116 GN=Tuba1b PE=3 SV=1	High	5
A0A8I6G6K9	*Elmo2*	Engulfment and cell motility 2 OS=Rattus norvegicus OX=10116 GN=Elmo2 PE=1 SV=1	High	2
A0A8I6GBC3	*Actr2*	Actin related protein 2 OS=Rattus norvegicus OX=10116 GN=Actr2 PE=1 SV=1	High	7
A0A8I6GLR6	*Prex1*	Phosphatidylinositol-3,4,5-trisphosphate-dependent Rac exchange factor 1 OS=Rattus norvegicus OX=10116 GN=Prex1 PE=4 SV=1	High	2
A0A8L2QIZ5	*C1qbp*	Complement component 1 Q subcomponent-binding protein, mitochondrial OS=Rattus norvegicus OX=10116 GN=C1qbp PE=1 SV=1	High	5
A0A8L2R7U3	*Tubb2b*	Tubulin beta chain OS=Rattus norvegicus OX=10116 GN=Tubb2b PE=3 SV=1	High	2
B2GV73	*Arpc3*	Actin-related protein 2/3 complex subunit 3 OS=Rattus norvegicus OX=10116 GN=Arpc3 PE=1 SV=1	High	4
D3ZRB3	*Rhof*	RCG21806 OS=Rattus norvegicus OX=10116 GN=Rhof PE=1 SV=2	High	2
D4A280	*Pak5*	Serine/threonine-protein kinase PAK 5 OS=Rattus norvegicus OX=10116 GN=Pak5 PE=1 SV=1	High	2
G3V6S0	*Sptbn1*	Spectrin beta chain OS=Rattus norvegicus OX=10116 GN=Sptbn1 PE=1 SV=4	High	91
P68035	*Actc1*	Actin, alpha cardiac muscle 1 OS=Rattus norvegicus OX=10116 GN=Actc1 PE=2 SV=1	High	3
Q5XI73	*Arhgdia*	Rho GDP-dissociation inhibitor 1 OS=Rattus norvegicus OX=10116 GN=Arhgdia PE=1 SV=1	High	8
Q7TT49	*Cdc42bpb*	Serine/threonine-protein kinase MRCK beta OS=Rattus norvegicus OX=10116 GN=Cdc42bpb PE=1 SV=1	High	24

Uniprot ID and gene name are presented for each protein together with its description and quality parameters of detection (confidence and nº of unique peptides detected).

Supplementary methods

Antibodies, chemicals and recombinant proteins, cell-penetrating peptides, primers, commercial assays, experimental models, recombinant DNAs and software are described in the Table S1, included as Supplementary material.

Experimental models

Animal procedures were performed following European Union Directive 2010/63/ EU and were approved by the CSIC and Comunidad de Madrid (Ref PROEX 276.6/20) ethics committees. Housing facilities were approved by Comunidad de Madrid (#ES 280790000188) and conform to official regulations. Animals had standard health and immune status and were looked after by professional caretakers daily. Male and female mice were kept in groups of up to five in standard IVC cages while 1–2 pregnant rats occupied standard cages, always containing bedding and nesting materials. Animals were under controlled lighting conditions (12 h light cycles), relative humidity and temperature. Irradiated food and water were provided *ad libitum.* We tried to minimize animal suffering and reduce the number of sacrificed animals.

Mice model of ischemia by microvascular photothrombosis to study in vivo excitotoxicity

This model of permanent focal ischemia mimics embolic or thrombotic occlusion of small arteries, which is frequently found in human stroke, and causes a focal brain damage with histological and MRI correlations to human patterns [1]. The procedure was as previously described [2] with some modifications. Male and female Balb/cOlaHsd mice (23–31 g; 10–16 weeks of age; Envigo Rms, Spain S.L.) were allowed to acclimatize to our facilities for at least 1 week after delivery, before ischemic induction. They were kept with the same cage mates, and daily examined to check their health status. Mice were anesthetized with isoflurane (5% for induction, 2% for maintenance in oxygen; Abbot Laboratories, Madrid, Spain) and then placed in a stereotaxic frame (Narishige Group, Tokyo, Japan). Body temperature was maintained at 36–37°C using a self-regulating heating blanket (Cibertec, Madrid, Spain). A midline scalp incision was made, the skull was exposed, and both Bregma and Lambda points were identified. A cold-light (Schott KL 2500 LCD; Schott Glass, Mainz, Germany) with a fibre optic bundle of 1.5 mm in diameter was centred using a micromanipulator on the right side, at 0.2 mm anterior and 2.0 mm lateral (+0.2 AP, +2 ML) relative to Bregma. Afterward, the photosensitive dye Rose Bengal (7.5 mg/ml, prepared in sterile saline; Sigma-Aldrich) was administered by retro-orbital injection of the venous sinus, for intravenous (i.v.) vascular access, to a body dose of 20 mg/kg. Five minutes later, the brain was illuminated (600 lms, 3,000 K) through the intact

skull for 10 min. Brain injury encompasses damage to vascular endothelium, platelet activation, and subsequent microvascular thrombotic occlusion of the irradiated region [3]. The areas underneath this stereotaxic position that result irradiated are the primary motor cortex and the primary somatosensory cortex (hindlimb and forelimb). Once completed surgery, the incision was sutured, and mice were allowed to recover.

For neuroprotection experiments, a single dose (3 nmol/g) of peptides TMyc or $TT1_{Ct}$ (> 95% purity; GenScript), solubilized as 2.5 mM solutions in 0.9% NaCl, we retro-orbitally injected 1 h after damage initiation. In order to improve their plasma stability, these peptides are N- and C-ter modified by, respectively, acetyl and amide groups. Mice were not subjected to other procedures before ischemia and were naive to drug or peptide treatment. Animals were randomly allocated to the experimental groups and the researchers doing the experiments were blind respect to treatment. There was not a previous estimation of sample size and experiments were independently carried out as indicated. Twenty-four hours after damage induction, animals completed the beam walking test and were subsequently sacrificed by cervical dislocation to measure the infarct volume, as indicated below. Before that, brains were sectioned into serial 1-mm-thick coronal slices using a mouse brain matrix (Stoelting, Wood Dale, IL, USA), and sections were completely stained with 2% TTC at room temperature to visualize the cortical infarcts. For immunohistochemistry, animals were deeply anesthetized 5 or 24 h after brain damage and intracardially perfused with cold PBS and 4% paraformaldehyde in PBS as indicated below.

Primary cultures of rat cortical neurons to study in vitro excitotoxicity

The dissected cerebral cortices of Wistar rat embryos (E18), both genders included, were mechanically dissociated in culture medium (Minimum Essential Medium, MEM, Life Technologies) followed by seeding of the cell suspension at a density of $1.1x10^6$ cells/ml, prepared in the same medium supplemented with 22.2 mM glucose, 0.1 mM glutamax, 5% foetal bovine serum (FBS), 5% donor horse serum (HS), 100 U/ml penicillin and 100 μg/ml streptomycin. Before seeding, the plates were treated overnight at 37°C with 100 μg/ml poly-L-lysine and 4 μg/ml laminin. The cells were incubated at 37°C in an atmosphere of 5% CO_2 and 95% humidity. Generally, glial growth was inhibited after 7 days *in vitro* (DIVs) by adding 10 μM 5 cytosine β-D-arabinofuranoside (AraC) and experimental treatments took place after 12 DIVs. Excitotoxicity was induced by incubation with NMDA (100 μM) and glycine (10 μM) for the indicated times. As indicated, cultures were preincubated with Tat-derived CPPs before NMDA treatment.

Primary cultures of rat cortical astrocytes

Dissected cerebral cortices obtained from Wistar rat E18 as before were mechanically dissociated in Hank's Balanced Salt Solution (HBSS, Thermo Fisher) containing $CaCl_2$ and $MgCl_2$, supplemented with 10 mM Hepes. The resulting cell suspension was then seeded in 75 cm² flasks pretreated with 100 µg/mL poly-L-lysine at a density of $4x10^6$ cells/ml in proliferation medium. This medium consisted of Dulbecco's Modified Eagle Medium/Nutrient Mixture F-12 with GlutaMAX (DMEM/F-12/GlutaMAX, Thermo Fisher), 10% FBS, 5% HS, 100 U/ml penicillin, 100 µg/ml streptomycin, 2% B27 supplement, 10 ng/ml epidermal growth factor (EGF), and 10 ng/ml basic fibroblast growth factor (bFGF).

After 4 DIVs, when cultures reached approximately 70–80% confluence, the medium was removed, and the flasks were vigorously washed with PBS to eliminate potential oligodendrocyte contaminants. The cells were then trypsinized using TrypLE Express (Thermo Fisher) and reseeded in flasks under the same conditions at a density of $3x10^6$ cells/ml in proliferation medium. Six days later, astrocytes were washed, trypsinized as before, and seeded at a density of $0.7x10^6$ cells/ml in poly-L-lysine-coated plates for 24 hours. After this period, the plates were washed with PBS, and the medium was replaced with differentiation medium consisting of DMEM/F-12/GlutaMAX, 10% FBS, 100 U/ml penicillin, and 100 µg/ml streptomycin.

Assessment of *in vivo* and *in vitro* excitotoxic injury

Measurement of infarct volume

Sections stained with 2% TTC, as before indicated, were subsequently scanned by both rostral and caudal sides. These images were analysed using ImageJ software by an observer blinded to experimental groups. After image calibration, delineated areas of ipsilateral and contralateral hemispheres, and the infarcted region (unstained area) were measured. Considering slices thickness, the corresponding volumes were calculated and corrected for oedema's effect, estimated by comparing total volumes of hemispheres. The corrected infarct volumes were expressed as percentage relative to the contralateral hemisphere, to correct for normal size differences between different animals. For each animal, the mean of results obtained for rostral and caudal sides was calculated.

Beam walking test

Motor coordination and balance were evaluated in mice right before and 24 h after the ischemic insult by measuring the number of contralateral hind paw slips in the beam walk apparatus.

Mice had to walk through a narrow beam (1 m × 1 cm × 1 cm) placed 50 cm above the tabletop, going from an aversive stimulus (60-W light bulb) to a black goal box with nesting material. Slips taking place in a previously selected central beam segment (50 cm long) were counted. Before damage induction, mice were allowed to cross the beam once, to get acquainted with the test, which they repeated 24 h after photothrombosis, immediately before sacrifice.

Assessment of neuronal injury in cortical cultures

Thiazolyl blue formazan (MTT) reduction assay was used to determine cell viability. At the end of the different treatments, 0.5 mg/ml MTT was added to the medium, and after 2 h of incubation at 37ºC the formazan salts formed were solubilized using DMSO. The results were quantified spectrophotometrically at 570 nm. As primary cortical cultures are formed by neurons and glial cells, we determined the magnitude of glia viability on the total values by applying 400 µM NMDA and 10 µM glycine to sister cultures 24 h before MTT assay. These conditions induce a complete neuronal death but no glial damage. Thus, after subtracting this absorbance value, the results only represent the viability of neurons present in cultures. Each independent experiments included technical triplicates for every treatment condition. In addition, a minimum of 4 completely independent experiments were performed and analysed as indicated in figure legends. When indicated, the provided viability data correspond to total cell values (neurons and glial cells.

Synthetic peptides

Synthetic cell-penetrating peptides (> 95% purity; GenScript) were used for treatment of primary cortical cultures and mice. All these CPPs contain a HIV-1 Tat sequence (11 aa) linked to a specific TrkB-T1 or c-Myc sequence as indicated (Table S1). Three of these peptides (Bio-TMyc, Bio-sTT1$_{Ct}$ and Bio-TT1$_{Ct}$) contain a biotin molecule at the N-ter, which serves several purposes: peptide labelling, stabilization, and competence to isolate proteins interacting with TrkB-T1 taking place inside cultured cells. In the other two peptides (TMyc and TT1$_{Ct}$), the N-ter is acetylated while all five CPPs have their C-ter amidated. The concentration of use is indicated for each experiment.

Pull down assays

Cultures incubated with biotinylated peptides and treated as indicated were lysed at 4°C in NP-40 lysis buffer (1% NP-40, 20 mM Tris HCl pH 8.0, 80 mM NaCl, 20 mM EDTA) containing protease and phosphatase inhibitors (Table S1), conditions that do not disrupt peptide-protein interactions. After establishing protein concentration (BCA Protein Assay Kit), equal protein

amounts were combined with streptavidin-agarose beads previously washed with NP-40 lysis buffer and incubated at room temperature for 1.5 h with agitation. Sedimented beads were washed 3 times with the NP-40 lysis buffer to remove possible non-specifically bounded proteins, and peptides and their interacting proteins were released by incubation at 50°C for 40 min in RIPA modified buffer (50 mM Tris HCl pH 8.0, 150 mM NaCl, 1% sodium deoxycholate, 1% NP-40) combined with protease and phosphatase inhibitors as before.

Proteomic analysis of Bio-sTT1$_{Ct}$-interacting proteins

Proteomic analysis was performed in ProstaR (1.34.6) software tool [4], using the protocol for protein-level analysis [5]. Proteomic abundances from 4 independent experiments were log-transformed and filtered following quality parameters: proteins present in at least >50% of one of the conditions and identified by at least 2 unique peptides. After, data was normalized by LOESS (locally estimated scatterplot smoothing, k=0.7), and POV (partially observed values) and MEC (missing in entire condition values) imputations were made using, respectively, KNN (k-nearest-neighbour k=10) and det.quantile (quantile imputation using quantile = 2,5). Differential analysis was performed using limma (3.16). Multiple comparison p-values where calibrated using slim algorithm and the FDR (false discovery rate) is indicated for each comparison. For PCA (principal component analysis), filtered, normalized and imputed data was used corrected for batch effect using removeBatchEffect {limma} function. First and second principal components are presented. For volcano plot representation ggplot2 (3.5.1) software was used, and for heatmap presentation ComplexHeatmap (2.18.0) software. Enrichment analysis for GO (Gene Ontology terms) and Reactome (Reactome.db) pathway analysis the string.db (12.0) tool was used.

Immunoblot analysis

Cells were lysed in RIPA buffer (50 mM Tris-HCl pH 8.0, 150 mM NaCl, 1% sodium deoxycholate, 1% NP-40, 0.1% SDS, 1 mM DTT), supplemented with protease and phosphatase inhibitors as above, for 30 min at 4ºC. Then, cell lysates were sonicated using a Bioruptor apparatus and centrifugated at 4ºC for 20 min at 10,000 rpm. The protein concentration in lysates was established with BCA Protein Assay Kit (Thermo Fisher) followed by 5 min denaturalization in SDS-sample buffer at 95º. Equal amounts of total lysates were resolved in Tris-Glycine SDS-PAGE and transferred to a nitrocellulose membrane (GE Healthcare) in 25 mM Tris HCl pH 8.3, 250 mM glycine and 10% methanol, using an electric current of 400 mA for 70 min. After transfer, membranes were stained with a Ponceau S solution to check for its efficacy. Then, membranes were blocked for 30 min with a 5% non-fat

dry milk solution in Tris Buffered Saline-Tween (TBS-T, 20 mM Tris HCl pH 7.5, 137 mM NaCl, 0.05% Tween-20) and incubated overnight at 4°C with primary antibodies. Next, membranes were washed with TBS-T and incubated with the appropriate anti-rabbit or anti-mouse peroxidase-conjugated secondary antibodies (Bethyl) for 1 h. To conclude, immunoreactivity was detected using Clarity Western ECL Blotting Substrate (BioRad) and band intensity was quantified by densitometric analysis (Photoshop, Adobe). Levels of the protein of interest were normalized using those of neuron-specific enolase (NSE) present in the same sample and expressed relative to values obtained in their respective controls, arbitrarily given a 100% value. NSE was used as a neuronal loading control since it is not affected by NMDA treatment. Exceptionally, levels of TrkB-T1 were normalized to those of ß-actin in the comparison of primary mixed cultures and those of astrocytes. Multiple independent experiments were carried out and quantitated as detailed in the figure legends.

Cell transfection and gene reporter assays

Plasmids contained minimal CREB or MEF2 response elements upstream firefly luciferase reporter gene (respectively, pCRE or pMEF2; see details in Supplementary Table 1). Primary cultures, without the AraC treatment, were transfected at 11 DIVs with 0.4 µg of plasmids using Lipofectamine 2000. DNA-liposomes complexes were prepared according to the manufacturer instructions in neurobasal medium (Thermo Fisher Scientific) with 1 mM glutamax (Gibco, Thermo Fisher Scientific) and added to the cell culture. After 2 h of transfection, this mix was replaced with previously collected conditioned medium and incubation proceeded to complete 24 h. Protein extracts were obtained using Passive Lysis Buffer (Promega, Cat# E1941) and luciferase activity was detected by a luminometer (GloMax 96 microplate luminometer, Promega), using REAP buffer (25 mM glycylglycine, 15 mM SO_4Mg, 4 mM EGTA, 15 mM potassium phosphate pH 7.8, 3.3 mM ATP, 1 mM DTT, 75 µM luciferin). Each experiment included technical quadruplicates for every condition, and 5-7 totally independent experiments were analysed as indicated.

RNA extraction and qPCR assay

Total RNA was extracted using QIAcube technology and treated with DNases before cDNA synthesis by the "High Capacity cDNA Reverse Transcription Kit" (Applied Biosystems). A 7900HT Fast real-time PCR system (Applied Biosystems) was used for SYBR green gene expression assays, with the primers indicated in Supplementary Table 1. PCR conditions were 10 min at 95ºC, followed by 40 cycles of 15 s at 95ºC and 60 s at 60ºC. For each independent experiment, we made a specific standard curve for every gene and technical triplicates were

prepared for every sample. Then, we performed a total of 5 completely independent experiments. Results were normalised to the levels of housekeeping genes as indicated in the "Methods" section.

Peptide visualization in primary cultures and immunocytochemistry

Primary cultures, seeded at half the concentration previously indicated, were grown for 13 DIVs on coverslips previously treated overnight at 37°C with poly-L-lysine and laminin as before. Next, cells were incubated with Bio-TMyc or Bio-sTT1$_{Ct}$ (25 μM, 1 h) or left untreated, and then fixed for 30 min with 4% paraformaldehyde in PBS. The fixed cells were washed several times with PBS to eliminate the remaining paraformaldehyde before they were blocked and permeabilized for 4 h with 4% goat serum and 0.5% Triton X-100 prepared in PBS. Afterwards coverslips were incubated overnight at 4°C with the corresponding primary antibodies, diluted in the blocking solution. After washing with PBS, the coverslips were incubated with the corresponding secondary antibodies together with Fluorescein Avidin D (200 μg/ml) for 1 h at room temperature and then 10 min with DAPI, to stain DNA. Finally, the samples were mounted in Prolong Diamond. The images were acquired using an inverted Zeiss LSM 710 laser confocal microscope (Jena, Germany) with a 40x or 63x Plan-Apochromatic oil immersion objective. Images correspond to single sections and were normalized for each colour separately and processed for presentation with ImageJ (NIH Image) and Fiji.

Peptide visualization and immunohistochemistry in brain cortex

For peptide visualization in undamaged or damaged brains, animals were deeply anesthetized and intracardially perfused with cold PBS and 4% paraformaldehyde in PBS. Brains were post-fixed in the same fixative at 4°C for 24 h and cryoprotected in 30% sucrose for 48 h at 4°C. Coronal frozen sections (30 μm thick) obtained using a cryostat (Leica, Heidelberg, Germany) were incubated in blocking solution (10% goat serum, 0.5% Triton X-100 in PBS) for 3 h at room temperature, followed by Fluorescein Avidin D (200 μg/ml), and DAPI (5 μg/ml) prepared in 4% goat serum for 1 h. When indicated, sections were incubated for 3 h with anti-NeuN in 4% goat serum right after blocking and, after washing, with Alexa Fluor 647-conjugated antibodies together with Fluorescein Avidin D and DAPI as before indicated. Finally, sections were mounted and dried on slides, and cover slipped with Prolong Diamond. Image acquisition was performed using an inverted laser confocal microscope as before with a 63x or 40x objective Plan-Apochromatic oil immersion objective. Images were processed as described and correspond to single sections. Background was subtracted using vehicle-injected animals.

Immunohistochemistry

The brains of animals injected one hour after the initiation of the photothrombotic damage with vehicle (saline), TMyc or $TT1_{Ct}$ (3 nmol/g) were processed and cryoprotected as before indicated. The duration of damage before sacrifice was 5 h (male and female) or 24 h (male). Coronal frozen sections were incubated with blocking solution as described in flotation for 1 h at room temperature and then overnight with primary antibodies diluted in 4% goat serum and 0.3% Triton X-100 in PBS at 4°C. After washing, slides were incubated 1 h with Alexa Fluor 546-conjugated goat anti-rabbit or AlexaFluor 647-conjugated goat anti-mouse secondary antibodies to visualize the primary antibodies and DAPI (5 µg/ml). Only for Iba1 staining, blocking solution was 2.5% BSA, 5% goat serum, 0.5% Triton X-100 in PBS, and sections were incubated with primary antibody 24 h at RT followed by washing and Alexa Fluor 546-conjugated goat anti-rabbit and DAPI incubation for 1 h. Sections were then washed in PBS, mounted on slides, dried for 15 min at 37°C on a hot plate, or air dried overnight and then cover slipped with DPX or Prolong Diamond. Cell Observed images were obtained with Zeiss microscope Observer.Z1 using a 10x Plan-Apochromatic objective. Confocal images were acquired with a 40x or 63x Plan-Apochromatic oil immersion objective and processed for presentation as above described.

References

1. Pevsner PH, Eichenbaum JW, Miller DC, Pivawer G, Eichenbaum KD, Stern A, et al. A photothrombotic model of small early ischemic infarcts in the rat brain with histologic and MRI correlation. J Pharmacol Toxicol Methods. 2001; 45: 227-33.

2. Tejeda GS, Esteban-Ortega GM, San Antonio E, Vidaurre OG, Díaz-Guerra M. Prevention of excitotoxicity-induced processing of BDNF receptor TrkB-FL leads to stroke neuroprotection. EMBO Mol Med. 2019; 11:e9950.

3. Watson BD, Dietrich WD, Busto R, Wachtel MS, Ginsberg MD. Induction of reproducible brain infarction by photochemically initiated thrombosis. Ann Neurol. 1985; 17: 497-504.

4. Wieczorek S, Combes F, Lazar C, Giai Gianetto Q, Gatto L, Dorffer A, et al. DAPAR & ProStaR: software to perform statistical analyses in quantitative discovery proteomics. Bioinformatics. 2017; 33: 135-6.

5. Wieczorek S, Combes F, Borges H, Burger T. Protein-Level Statistical Analysis of Quantitative Label-Free Proteomics Data with ProStaR. Methods Mol Biol. 2019; 1959: 225-46.
